# Single-nucleus transcriptomics uncovers a geroprotective role of YAP in primate gingival aging

**DOI:** 10.1093/procel/pwae017

**Published:** 2024-04-05

**Authors:** Qinchao Hu, Bin Zhang, Yaobin Jing, Shuai Ma, Lei Hu, Jingyi Li, Yandong Zheng, Zijuan Xin, Jianmin Peng, Si Wang, Bin Cheng, Jing Qu, Weiqi Zhang, Guang-Hui Liu, Songlin Wang

**Affiliations:** State Key Laboratory of Membrane Biology, Institute of Zoology, Chinese Academy of Sciences, Beijing 100101, China; Hospital of Stomatology, Guanghua School of Stomatology, Guangdong Provincial Key Laboratory of Stomatology, Sun Yat-sen University, Guangzhou 510060, China; State Key Laboratory of Membrane Biology, Institute of Zoology, Chinese Academy of Sciences, Beijing 100101, China; Key Laboratory of Organ Regeneration and Reconstruction, Institute of Zoology, Chinese Academy of Sciences, Beijing 100101, China; University of Chinese Academy of Sciences, Beijing 100049, China; State Key Laboratory of Membrane Biology, Institute of Zoology, Chinese Academy of Sciences, Beijing 100101, China; Key Laboratory of Organ Regeneration and Reconstruction, Institute of Zoology, Chinese Academy of Sciences, Beijing 100101, China; Institute for Stem Cell and Regeneration, Chinese Academy of Sciences, Beijing 100101, China; Beijing Institute for Stem Cell and Regenerative Medicine, Beijing 100101, China; International Center for Aging and Cancer, Hainan Medical University, Haikou 571199, China; State Key Laboratory of Membrane Biology, Institute of Zoology, Chinese Academy of Sciences, Beijing 100101, China; Key Laboratory of Organ Regeneration and Reconstruction, Institute of Zoology, Chinese Academy of Sciences, Beijing 100101, China; Institute for Stem Cell and Regeneration, Chinese Academy of Sciences, Beijing 100101, China; Beijing Institute for Stem Cell and Regenerative Medicine, Beijing 100101, China; Aging Biomarker Consortium, Beijing 100101, China; Salivary Gland Disease Center and Beijing Key Laboratory of Tooth Regeneration and Function Reconstruction, Beijing Laboratory of Oral Health and Beijing Stomatological Hospital, Capital Medical University, Beijing 100050, China; State Key Laboratory of Membrane Biology, Institute of Zoology, Chinese Academy of Sciences, Beijing 100101, China; Key Laboratory of Organ Regeneration and Reconstruction, Institute of Zoology, Chinese Academy of Sciences, Beijing 100101, China; University of Chinese Academy of Sciences, Beijing 100049, China; Institute for Stem Cell and Regeneration, Chinese Academy of Sciences, Beijing 100101, China; Beijing Institute for Stem Cell and Regenerative Medicine, Beijing 100101, China; Aging Biomarker Consortium, Beijing 100101, China; State Key Laboratory of Stem Cell and Reproductive Biology, Institute of Zoology, Chinese Academy of Sciences, Beijing 100101, China; Key Laboratory of Organ Regeneration and Reconstruction, Institute of Zoology, Chinese Academy of Sciences, Beijing 100101, China; University of Chinese Academy of Sciences, Beijing 100049, China; State Key Laboratory of Membrane Biology, Institute of Zoology, Chinese Academy of Sciences, Beijing 100101, China; Key Laboratory of Organ Regeneration and Reconstruction, Institute of Zoology, Chinese Academy of Sciences, Beijing 100101, China; Institute for Stem Cell and Regeneration, Chinese Academy of Sciences, Beijing 100101, China; Beijing Institute for Stem Cell and Regenerative Medicine, Beijing 100101, China; Hospital of Stomatology, Guanghua School of Stomatology, Guangdong Provincial Key Laboratory of Stomatology, Sun Yat-sen University, Guangzhou 510060, China; Advanced Innovation Center for Human Brain Protection and National Clinical Research Center for Geriatric Disorders, Xuanwu Hospital Capital Medical University, Beijing 100053, China; Aging Translational Medicine Center, International Center for Aging and Cancer, Beijing Municipal Geriatric Medical Research Center, Xuan Wu Hospital, Capital Medical University, Beijing 100053, China; Aging Biomarker Consortium, Beijing 100101, China; Hospital of Stomatology, Guanghua School of Stomatology, Guangdong Provincial Key Laboratory of Stomatology, Sun Yat-sen University, Guangzhou 510060, China; State Key Laboratory of Stem Cell and Reproductive Biology, Institute of Zoology, Chinese Academy of Sciences, Beijing 100101, China; Key Laboratory of Organ Regeneration and Reconstruction, Institute of Zoology, Chinese Academy of Sciences, Beijing 100101, China; University of Chinese Academy of Sciences, Beijing 100049, China; Institute for Stem Cell and Regeneration, Chinese Academy of Sciences, Beijing 100101, China; Beijing Institute for Stem Cell and Regenerative Medicine, Beijing 100101, China; Aging Biomarker Consortium, Beijing 100101, China; CAS Key Laboratory of Genomic and Precision Medicine, Beijing Institute of Genomics, Chinese Academy of Sciences and China National Center for Bioinformation, Beijing 100101, China; University of Chinese Academy of Sciences, Beijing 100049, China; Institute for Stem Cell and Regeneration, Chinese Academy of Sciences, Beijing 100101, China; Aging Biomarker Consortium, Beijing 100101, China; State Key Laboratory of Membrane Biology, Institute of Zoology, Chinese Academy of Sciences, Beijing 100101, China; Key Laboratory of Organ Regeneration and Reconstruction, Institute of Zoology, Chinese Academy of Sciences, Beijing 100101, China; University of Chinese Academy of Sciences, Beijing 100049, China; Institute for Stem Cell and Regeneration, Chinese Academy of Sciences, Beijing 100101, China; Beijing Institute for Stem Cell and Regenerative Medicine, Beijing 100101, China; Advanced Innovation Center for Human Brain Protection and National Clinical Research Center for Geriatric Disorders, Xuanwu Hospital Capital Medical University, Beijing 100053, China; Aging Translational Medicine Center, International Center for Aging and Cancer, Beijing Municipal Geriatric Medical Research Center, Xuan Wu Hospital, Capital Medical University, Beijing 100053, China; Aging Biomarker Consortium, Beijing 100101, China; Salivary Gland Disease Center and Beijing Key Laboratory of Tooth Regeneration and Function Reconstruction, Beijing Laboratory of Oral Health and Beijing Stomatological Hospital, Capital Medical University, Beijing 100050, China; Department of Biochemistry and Molecular Biology, School of Basic Medical Sciences, Capital Medical University, Beijing 100069, China

**Keywords:** single-nucleus RNA-sequencing, primate, gingiva, aging, YAP

## Abstract

Aging has a profound impact on the gingiva and significantly increases its susceptibility to periodontitis, a worldwide prevalent inflammatory disease. However, a systematic characterization and comprehensive understanding of the regulatory mechanism underlying gingival aging is still lacking. Here, we systematically dissected the phenotypic characteristics of gingiva during aging in primates and constructed the first single-nucleus transcriptomic landscape of gingival aging, by which a panel of cell type-specific signatures were elucidated. Epithelial cells were identified as the most affected cell types by aging in the gingiva. Further analyses pinpointed the crucial role of YAP in epithelial self-renew and homeostasis, which declined during aging in epithelial cells, especially in basal cells. The decline of YAP activity during aging was confirmed in the human gingival tissues, and downregulation of *YAP* in human primary gingival keratinocytes recapitulated the major phenotypic defects observed in the aged primate gingiva while overexpression of YAP showed rejuvenation effects. Our work provides an in-depth understanding of gingival aging and serves as a rich resource for developing novel strategies to combat aging-associated gingival diseases, with the ultimate goal of advancing periodontal health and promoting healthy aging.

## Introduction

The oral mucosa, an important barrier tissue in the human mouth, includes the gingiva, a masticatory tissue that surrounds the teeth and with unique histological and molecular characteristics relative to other oral mucosae (such as the buccal mucosa, the inside lining of the cheeks) (Williams et al., 2021). Aging has a profound influence on the gingiva, reflected as an increased incidence of multiple gingiva-related diseases, the most representative of which is periodontitis. As a highly prevalent chronic inflammatory disease that occurs in the tooth-supporting tissues, periodontitis affects both the gingiva and the alveolar bone. The prevalence of periodontitis in elderly ≥65 years of age can be as high as 96.4% (Eke et al., 2016) and causes destruction of periodontal tissues and loss of teeth, but more importantly, is also associated with increased risks of a series of systemic diseases, such as Alzheimer’s disease, rheumatoid arthritis, cardiovascular disease, inflammatory bowel disease, etc. (Hajishengallis and Chavakis, 2021), and an increased risk of all-cause and cause-specific mortality (Romandini et al., 2021). At present, periodontitis is known to be closely related to extrinsic factors such as microbial dysbiosis, dental plaque and calculus accumulation, smoking, etc. (Kinane et al., 2017). However, how intrinsic factors, such as aging, affect periodontal tissues and increase the susceptibility of periodontitis is largely unknown. Therefore, exploring the effects of aging on gingiva is of great scientific and clinical significance, as such studies can help inform how to maintain periodontal health and prevent aging-related diseases such as periodontitis, as well as periodontitis-associated systemic comorbidities.

Gingiva consists of two layers: the surface stratified squamous epithelium and the deeper lamina propria. The gingival epithelium is composed of four layers: stratum basale, stratum spinosum, stratum granulosum, and stratum corneum. The junctional epithelium is an epithelial component that attaches directly to the tooth surface (Groeger and Meyle, 2019). The major components of the lamina propria are collagen fibers, which are produced by fibroblasts, holding the gingiva in place around the teeth and providing the strength required to withstand chewing pressure. The lamina propria also houses multiple types of immune cells and endothelial cells (Bartold et al., 2000). To date, reported histological changes in the gingiva during aging are inconclusive (Chen et al., 2022; Lamster et al., 2016), and a systematic dissection of gingiva aging at single-cell resolution is lacking. In multiple other organs, the heterogeneous process of aging has been investigated using emerging single-cell/nucleus RNA sequencing (scRNA-seq/snRNA-seq) techniques to elucidate transcriptional alterations underlying aging at cell-type-specific resolution (Huang et al., 2023; Jing et al., 2023; Li et al., 2021; Ma et al., 2021, 2023; Wang et al., 2020, 2021; Yang et al., 2023; Zhang et al., 2020b, 2021, 2023a, 2023b; Zou et al., 2021). Given the heterogeneity and dynamics of gingival tissue, unraveling its cell type-specific gene expression signatures is an important step towards deepening our understanding of gingival aging.

Non-human primates (NHPs) are genetically and biologically close to humans (Roth et al., 2004) and have a similar dental and periodontal structure to that of humans (Hlusko et al., 2016), thus representing an ideal model for studying primate gingival aging. In this study, we portray the phenotypic characteristics of NHP gingival aging and generate a single-nucleus transcriptomic atlas, in which we identified a panel of regulatory factors, including YAP, with distinct and unique gene expression signatures. We also reveal and experimentally validate *in vitro* that compromised YAP activity causally triggered primate gingival aging while overexpression of YAP showed rejuvenation effects. Our work provides a comprehensive understanding of gingival aging at a single-cell level, serving as a resource for developing new therapeutic strategies to combat aging-related gingival diseases.

## Results

### Imaging and histological features of periodontal aging in NHPs

**Figure 1.**
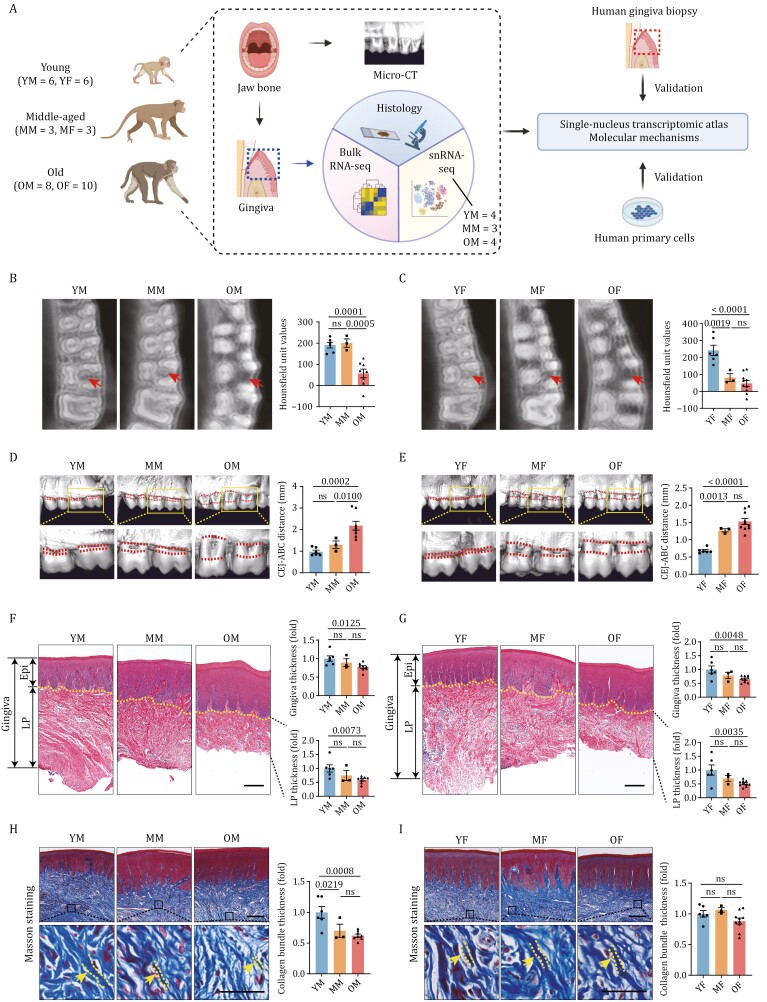
Imaging and histological features of periodontal aging in cynomolgus monkeys. (A) Flow chart of the experimental design of this study. (B and C) Bone density analysis of the alveolar bone by micro-CT. Representative images are shown on the left and quantitative data are shown as means ± SEM on the right. The red arrow indicates the representative area. (D and E) Micro-CT visualization of periodontal bone loss during aging. Representative images are shown on the left and quantitative data are shown as means ± SEM on the right. The red dotted line indicates the distance between the cementoenamel junction and alveolar bone crest (CEJ-ABC distance). (F and G) Hematoxylin and eosin (H&E) staining of gingival tissues. Representative images are shown on the left and quantitative data are shown as means ± SEM on the right. The yellow dotted line indicates the boundary between the epithelium and lamina propria. Epi, epithelium; LP, lamina propria. Scale bars, 200 μm. (H and I) Masson’s trichrome staining of gingival tissues. Representative images are shown on the left and quantitative data are shown as means ± SEM on the right. The yellow arrow and dotted line indicate the representative area of thickness of the collagen bundles. Scale bars, 200 μm and 50 μm (zoomed-in images). YM, young male (*n* = 6); MM, middle-aged male (*n* = 3); OM, old male (*n* = 8); YF, young female (*n* = 6); MF, middle-aged female (*n* = 3); OF, old female (*n* = 10). The *P*-values are indicated in the graphs, and ns indicates not significant.

To dissect the phenotypic and molecular characteristics of primate periodontal aging, we obtained periodontal tissues from 12 young (4–5 years old, equivalent to ~16 years old in human age), 6 middle-aged (10–12 years old, equivalent to ~40 years old in human age), and 18 aged (16–19 years old, equivalent to ~70 years old in human age) cynomolgus monkeys (Figs. 1A and S1A). According to age and sex, the monkeys were divided into young male (YM, *n* = 6), young female (YF, *n* = 6), middle-aged male (MM, *n* = 3), middle-aged female (MF, *n* = 3), old male (OM, *n* = 8), and old female (OF, *n* = 10) groups. First, we measured the bone density of the alveolar bone by micro-computed tomography (Micro-CT), and validated that bone density decreased with age (Fig. 1B and 1C). Further, the image of the alveolar bone was reconstructed in three dimensions to observe the periodontal bone loss during aging. The results revealed that the degree of periodontal bone loss constantly increased with age (Fig. 1D and 1E). However, we noted that periodontal bone loss was more pronounced in males (2.18 ± 0.20 mm in the OM group) than in females (1.53 ± 0.09 mm in the OF group), suggesting that the effect of aging on periodontal tissue is more prominent in males. As periodontal bone loss is usually caused by inflammation in the gingiva (Kinane et al., 2017), we took a closer look at histological changes in the gingiva during aging. Through HE staining analysis, we found that the overall thickness of the gingiva thinned markedly during aging, both in males and females (Fig. 1F and 1G). We also noted that the decreased thickness mainly occurred in the lamina propria (Fig. 1F and 1G), but not in the epithelial layer (Fig. S1B and S1C). When we conducted Masson staining to analyze the collagen fibers in the lamina propria, we found that the thickness of the collagen bundles was markedly decreased, whereas the collagen density was not (Figs. 1H, 1I, S1D and S1E). Notably, this alteration was present in males but not in females (Fig. 1H and 1I), again indicating that aging-associated features in periodontal tissue may be more prominent in males.

### Aging-associated indicators in primate gingiva

**Figure 2.**
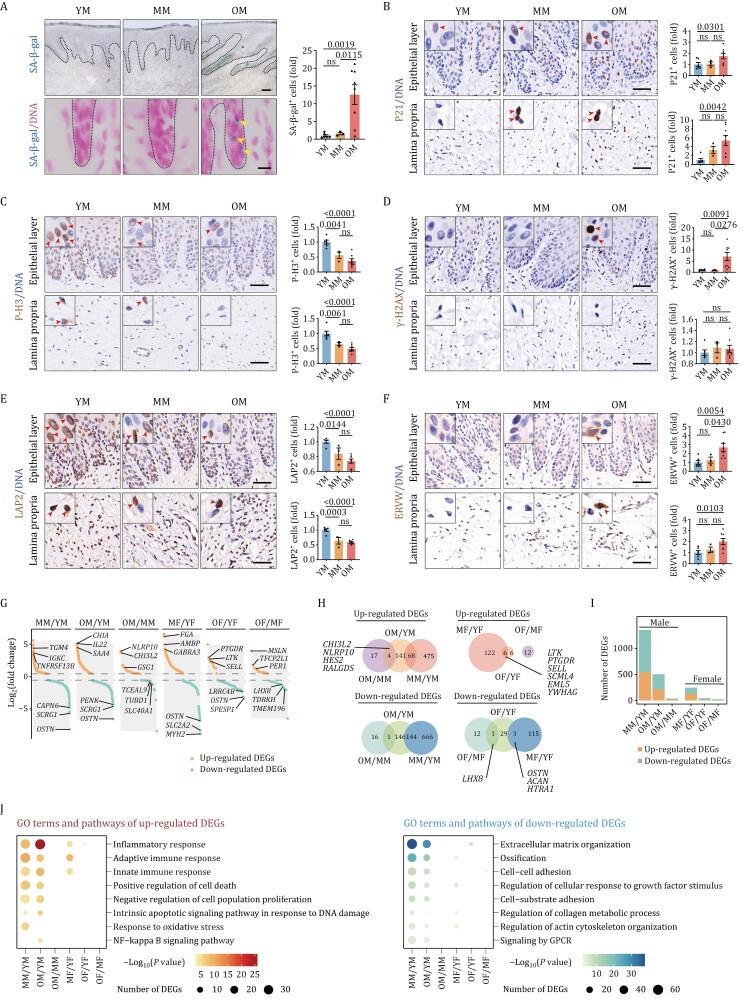
Aging-associated indicators in primate gingiva. (A) SA-β-gal staining of male monkey gingival tissues (upper) and counterstaining with nuclear fast red (lower). Representative images are shown on the left and quantitative data are shown as means ± SEM on the right. The yellow arrows indicate the SA-β-gal-positive cells. Scale bars, 200 μm (upper) and 10 μm (lower). (B–F) Immunohistochemistry staining of P21 (B), P-H3 (C), γ-H2AX (D), LAP2 (E), and ERVW (F) in gingival tissues of male monkeys. Representative images are shown on the left and quantitative data are shown as means ± SEM on the right. The red arrows indicate the positive cells. Scale bars, 50 μm. (G) The dot plots of differentially expressed genes (DEGs) in the gingiva of monkeys during aging indicated by bulk RNA sequencing. The orange dots indicate upregulated genes, the cyan dots indicate downregulated genes, and the gray blocks indicate the range of fold-change of DEGs. The top 3 genes with identified gene names are annotated. (H) Venn diagrams showing the shared upregulated (upper) and downregulated (lower) DEGs during aging. (I) The number of DEGs in the gingiva of monkeys during aging. (J) Representative GO terms and pathways of the upregulated (left) and downregulated (right) DEGs in gingival tissues of monkeys during aging. YM, young male (*n* = 6); MM, middle-aged male (*n* = 3); OM, old male (*n* = 8); YF, young female (*n* = 6); MF, middle-aged female (*n* = 3); OF, old female (*n* = 10). The *P*-values are indicated in the graphs and ns indicates not significant.

Next, we analyzed the gingiva with a panel of aging-related markers. In the male monkeys, the proportion of senescence-associated β-galactosidase (SA-β-gal) positive cells increased significantly during aging, and the positive signals were mainly located in the epithelial layer (Fig. 2A), implying the susceptibility of epithelial cells to aging. The expression of P21, another classic marker of senescence (Aging Biomarker et al., 2023; Cai et al., 2022), was also gradually increased during aging, both in the epithelial layer and lamina propria (Fig. 2B). Consistently, phospho-Histone H3 (P-H3) and PCNA, markers of cell proliferation, were gradually decreased during aging both in the epithelial layer and lamina propria (Figs. 2C and S2A). We also found that various markers of genomic and epigenomic instability, which are all recognized as hallmarks of aging (Wang et al., 2022; Wu et al., 2023; Zhang et al., 2020a), were dysregulated in the aged gingiva, including γ-H2AX (Fig. 2D), LAP2 (Fig. 2E), H3K9me3 (Fig. S2B), and Lamin B1 (Fig. S2C). In recent works, we reported that aging-associated epigenetic derepression could lead to the resurrection of endogenous retroviruses (ERVs) that drive the aging process (Liu et al., 2023; Wang et al., 2023b; Zhang et al., 2023a). Consistently, we found that the expression of ERVW increased during aging (Fig. 2F). This was also the case in the female monkey gingival samples but the changes were not as evident as in males (Fig. S2D–J).

To generate a global view of the transcriptional response to aging, we conducted bulk RNA sequencing (bulk RNA-seq) of the gingival tissues to identify differentially expressed genes (DEGs, adjusted *P* value ≤ 0.05 and |Log_2_FC| ≥ 0.5) between MM/YM groups, OM/YM groups, OM/MM groups, MF/YF groups, OF/YF groups, and OF/MF groups (Fig. 2G). Among the top-upregulated DEGs during aging, we identified several genes involved in immunity, inflammatory response, and antimicrobial defense, such as *IGKC*, *TNFRSF13B*, *IL22*, and *NLRP10* (Fig. 2G). Interestingly, we found that *FGA*, a gene that plays an important role in periodontitis (Silva et al., 2021), was substantially elevated during aging (Fig. 2G). In comparison, the top-downregulated DEGs were mainly associated with bone formation and regeneration, including *OSTN* and *SCRG1* (Fig. 2G), consistent with the increased periodontal bone loss during aging (Fig. 1D and 1E). When we analyzed overlapping DEGs between different age groups, we identified *NLRP10*, *OSTN*, and *SCRG1* (Fig. 2G and 2H). By comparing the number of DEGs, we found less aging-related DEGs in females relative to males (Fig. 2I), suggesting that the effect of aging in gingiva is more profound in males.

To further analyze the biological functions of aging-related DEGs in the gingiva, we performed an enrichment analysis. The age-upregulated genes were mainly enriched in the Gene Ontology (GO) terms inflammation and immune response (Fig. 2J), consistent with our previous findings identifying chronic inflammation as a common feature of primate organ aging (Jing et al., 2023; Ma et al., 2021; Yang et al., 2023; Zhang et al., 2021, 2023a; Zou et al., 2021). In addition, pathways involved in genomic instability and proliferation arrest were also upregulated, such as response to DNA damage, response to oxidative stress, and negative regulation of cell population proliferation (Fig. 2J), in line with upregulated γ-H2AX (Fig. 2D) and downregulated P-H3 (Fig. 2C) in staining analysis. In comparison, downregulated aging-related DEGs were mainly enriched for ossification, extracellular matrix organization, and regulation of collagen metabolic process (Fig. 2J), consistent with the loss of periodontal bone in the imaging analysis (Fig. 1D and 1E) and the thinning of lamina propria and collagen bundles in the histological examination (Fig. 1F–I). Additionally, pathways related to the epithelial barrier, such as cell–cell adhesion and cell–substrate adhesion, were also downregulated during aging (Fig. 2J). Given that elevated immunoinflammatory responses and epithelial barrier dysfunction are important pathologic mechanisms of periodontitis (Moutsopoulos and Konkel, 2018), these aging-related changes likely create a favorable environment for development of periodontitis.

### Construction of a single-nucleus transcriptomic atlas of the primate gingiva

**Figure 3.**
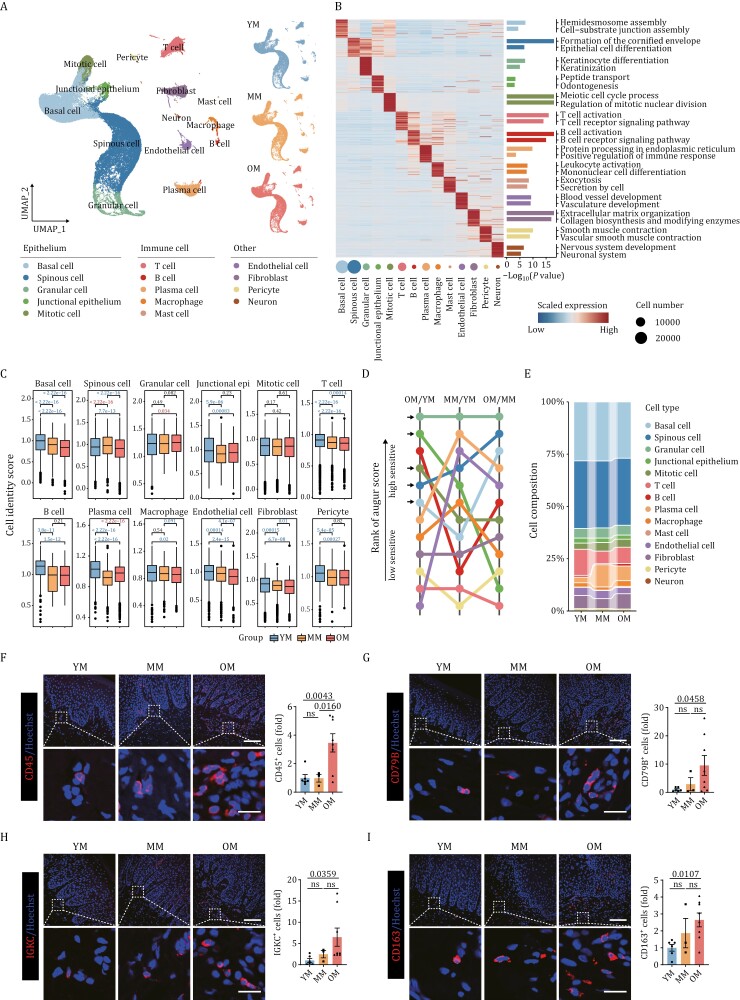
Single-nucleus transcriptomic atlas of the primate gingiva during aging. (A) Uniform manifold approximation and projection (UMAP) plots showing the cell types of monkey gingiva identified in single-nucleus RNA sequencing. Left, UMAP plots showing the 14 cell types of monkey gingiva. Right, UMAP plots showing the distribution of different cell types during aging. YM, young male (*n* = 4); MM, middle-aged male (*n* = 3); OM, old male (*n* = 4). (B) Heatmap showing the expression profiles of the top 50 marker genes of each cell type in the monkey gingiva with their enriched functional annotations on the right. (C) Box plot showing the cell identity score of each cell type in the monkey gingiva during aging. The *P*-values are indicated in the graphs, with the blue letter representing a significant downregulation during aging, the red letter representing a significant upregulation, and the black letter representing not significant. (D) Augur analysis showing prioritization of the most responsive cell types during gingival aging. The black arrows indicate epithelial cells. (E) Cell proportion of each cell type in the monkey gingiva during aging. (F–I) Immunofluorescence staining of CD45 (F), CD79B (G), IGKC (H), and CD163 (I) in gingival tissues of monkeys. Representative images are shown on the left and quantitative data are shown as means ± SEM on the right. The *P*-values are indicated in the graphs and ns indicates not significant. YM: *n* = 6; MM: *n* = 3; OM: *n* = 8. Scale bars, 100 μm and 20 μm (zoomed-in images).

To further investigate cell-type-specific alterations in the primate gingiva associated with aging, we conducted single-nucleus RNA sequencing (snRNA-seq). Given that such changes were more prominent in males, we performed snRNA-seq in the male gingiva, including 4 YM, 3 MM, and 4 OM samples. After stringent filtration, 85,730 qualified single nuclei were retained for subsequent analyses (Fig. S3A). Uniform Manifold and Projection (UMAP) algorithm analysis then identified a total of 14 cell types based on their specific markers (Caetano et al., 2021; Qian et al., 2021; Williams et al., 2021; Zou et al., 2021), including basal cells (*KRT5*^+^, *KRT15*^+^, and *COL17A1*^+^), spinous cells (*DSG1*^*+*^ and *CDH1*^*+*^), granular cells (*TGM3*^*+*^), junctional epithelium (*FDCSP*^*+*^), mitotic cells (*MKI67*^*+*^ and *TOP2A*^*+*^), T cells (*CD247*^*+*^, *CD3E*^*+*^, and *CD3G*^*+*^), B cells (*MS4A1*^*+*^ and *CD19*^*+*^), plasma cells (*IGKC*^*+*^ and *JCHAIN*^*+*^), macrophages (*CD74*^+^, *CD163*^+^, and *CSF1R*^*+*^), mast cells (*MS4A2*^*+*^ and *CPA3*^*+*^), endothelial cells (*PECAM1*^+^, *VWF*^+^, and *FLT1*^*+*^), fibroblasts (*DCN*^+^, *PDGFRA*^+^, *COL1A1*^+^, and *COL3A1*^*+*^), pericytes (*RGS5*^*+*^ and *PDGFRB*^*+*^), and neurons (*NRXN1*^*+*^) (Figs. 3A, S3B and S3C). Functional enrichment analysis of the top 50 marker genes of each cell type revealed unique transcriptional features relevant to their distinct physiological functions (Fig. 3B). For example, basal cell marker genes were associated with hemidesmosome assembly and cell–substrate junction assembly, while the fibroblast marker genes were associated with extracellular matrix organization and collagen biosynthesis (Fig. 3B).

Loss of cell identity is a common feature of cellular aging (Huang et al., 2023; Yang et al., 2023), consistent with our results showing that compromised cell identity was evident in the majority of cell types in gingiva showed, and most pronounced in basal cells (Fig. 3C). By performing Augur analysis (Skinnider et al., 2021), a method to prioritize the cell types most responsive to biological perturbations in single-cell data, we found that epithelial cells were the cell types most affected by aging (Fig. 3D). For the cell composition and proportion, we found that multiple immune cells tended to increase with age (Fig. 3E). Therefore, we detected the expression of CD45^+^ immune cells by immunofluorescence (IF) staining, and found that the infiltration of immune cells in gingival tissues increased substantially during aging (Fig. 3F), consistent with the GO term analysis detecting increased immune activation in bulk RNA-seq (Fig. 2J). Specifically, CD79B^+^ B cells (Fig. 3G), IGKC^+^ plasma cells (Fig. 3H), and CD163^+^ macrophages (Fig. 3I) all increased during aging.

### Characterization of the aging-associated cellular and molecular profiles of the primate gingiva

**Figure 4.**
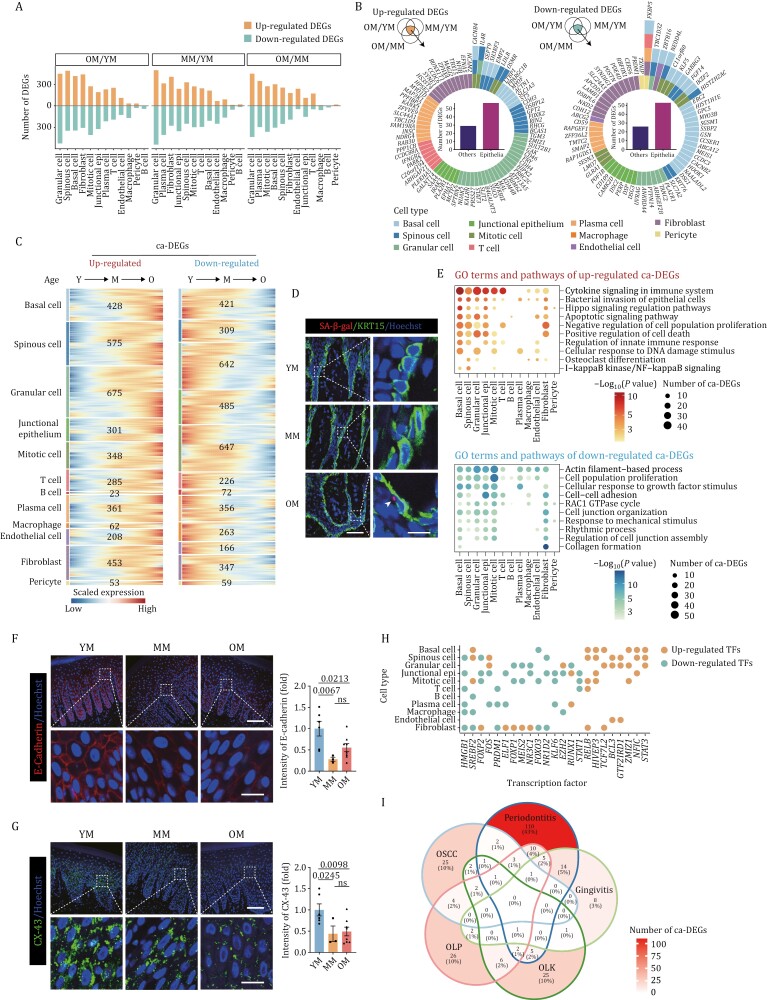
Aging-associated cellular and molecular characteristics of the primate gingiva. (A) The number of differentially expressed genes (DEGs) across different cell types in the monkey gingiva between different groups. YM, young male; MM, middle-aged male; OM, old male. (B) Radial plots showing the constant upregulated (left) and downregulated (right) DEGs which are overlapped in the Venn diagrams. The color keys indicate different cell types. The bar charts showing the number of DEGs in different cell types. (C) Heatmaps showing the upregulated (left) and downregulated (right) ca-DEGs across different cell types in the monkey gingiva. The numbers of ca-DEGs are annotated on the heatmaps. ca-DEGs, chronological age-DEGs; Y, young; M, middle-aged; O, old. (D) Representative image of co-staining of SA-β-gal and KRT15 in gingival tissues of monkeys. The images are transformed and merged using ImageJ. Scale bars, 50 μm and 10 μm (zoomed-in images). (E) Dot plots showing the representative shared GO terms of the age-dependent upregulated (upper) and downregulated (lower) DEGs across different cell types in the monkey gingiva. (F and G) Immunofluorescence staining of E-cadherin (F) and CX-43 (G) in gingival tissues of monkeys. Representative images are shown on the left and quantitative data are shown as means ± SEM on the right. The *P*-values are indicated in the graphs and ns indicates not significant. YM: *n* = 6; MM: *n* = 3; OM: *n* = 8. Scale bars, 100 μm and 20 μm (zoomed-in images). (H) Dot plots showing common upregulated (red) and downregulated (blue) transcription factors shared by at least three cell types in the monkey gingiva. (I) Venn diagram showing the association between ca-DEGs and aging-related diseases in the gingiva. OLK, oral leukoplakia; OLP, oral lichen planus; OSCC, oral squamous cell carcinoma.

To deepen our understanding of the transcriptomic landscape of primate gingival aging, we next analyzed age-associated DEGs (averaged Log_2_|FC| > 0.25 and adjusted *P* values < 0.05) between OM/YM groups, MM/YM groups, and OM/MM groups, respectively, across different cell types. We observed the largest numbers of DEGs in epithelial cells, such as granular cells, spinous cells, and basal cells (Fig. 4A), and that across the different age groups, consistently upregulated or downregulated DEGs were mainly present in epithelial cells (Fig. 4B). These results indicate that epithelial cells are more susceptible to aging, consistent with the staining results that mainly detected SA-β-gal activity in the epithelial layer (Fig. 2A). To further identify DEGs that were constantly increased or decreased during aging, we aligned samples by chronological age and clustered the DEGs (ca-DEGs) by their expression patterns (Zou et al., 2021). In this manner, we identified age-dependent upregulated and downregulated ca-DEGs in different cell types (Fig. 4C). The largest numbers of ca-DEGs were still observed in several types of epithelial cells (Fig. 4C), consistent with their known susceptibility to aging. Indeed, co-staining of SA-β-gal and KRT15 indicated that the senescent cells were mainly located in basal cells (Fig. 4D).

Next, we performed functional annotation enrichment analysis to investigate the molecular pathways most affected by aging across different cell types. First, upregulated ca-DEGs converged in several pathways associated with immune and inflammation response, such as cytokine signaling in immune system, regulation of innate immune response, and I-kappaB kinase/NF-kappaB signaling (Fig. 4E). GO terms associated with genomic instability and cell death, such as cellular response to DNA damage stimulus, apoptotic signaling pathway, and positive regulation of cell death, were also enriched in upregulated ca-DEGs across different cell types (Fig. 4E). As the gingiva is an important oral barrier, a breakdown of the gingival barrier enables the invasion of pathogenic bacteria and subsequent development of periodontitis (Vitkov et al., 2023). Here, we found that the upregulated ca-DEGs were associated with genes involved in bacterial invasion of epithelial cells, while the downregulated ca-DEGs were enriched in cell-cell adhesion, cell junction organization, and regulation of cell junction assembly (Fig. 4E), consistent with aging-associated breakdown of the gingival barrier. Consistently, IF analysis identified that the epithelial barrier-related proteins, E-cadherin and CX-43, declined significantly during aging (Fig. 4F and 4G). In addition, we also analyzed pathway enrichment based on DEGs identified between different age groups, and the results were consistent with that in ca-DEGs (Fig. S4A). Moreover, cell-type-specific ca-DEGs analysis revealed that DNA damage and Hippo signaling pathways were mainly upregulated in basal cells, while bacterial invasion was mainly upregulated in granular cells and junctional epithelium, and keratinocyte differentiation was upregulated in spinous cells and granular cells (Fig. S4B). Other cell types, such as fibroblasts, were mainly associated with a decline in extracellular matrix organization, collagen biosynthesis, collagen fibrils assembly, and ossification (Fig. S4B). In immune cells, including B cells, T cells, plasma cells, and macrophages, the ca-DEGs were mainly enriched in cell chemotaxis, leukocyte activation, and the release of inflammatory factors (Fig. S4B).

To further explore the core transcription factors (TFs) governing ca-DEGs, we performed single-cell regulatory network inference and clustering (SCENIC) analysis to construct a transcriptional regulatory network across different cell types (Fig. 4H). We found that TFs involved in the activation of the NF-κB pathway and inflammatory response, such as *RELB* (Sun, 2011) and *BCL3* (Wang et al., 2017), were upregulated during aging across several cell types. *STAT3* (Zou et al., 2020), a well-known transcriptional regulator of immune responses and tumorigenesis, was also upregulated during aging. For the age-downregulated TFs, we found that *HMGB1*, a conserved non-histone chromatin-associated protein with important roles in regulating the tertiary structure of chromatin and maintaining genome stability (Lou et al., 2021), was downregulated across cell types. More importantly, several genes reported as geroprotector in primates, including *KLF6* (in the skin) (Zou et al., 2021), *FOXO3* (in the artery and skeletal muscle) (Jing et al., 2023; Zhang et al., 2020b), and *FOXP1* (in the heart) (Zhang et al., 2023b), were found to decline during aging, especially in epithelial cells. Together, dysregulation of these core TFs is likely contributing to the process of primate gingival aging by modulating a series of target genes.

To understand the dynamic cell communication during aging, we constructed a cell–cell interaction network across cell types. Strong cell–cell interactions existed mainly between epithelial cells and epithelial cells, and between epithelial cells and fibroblasts (Fig. S4C). However, these interactions decreased distinctly during aging (Fig. S4D). Further, we analyzed these declined ligand–receptor pairs and found that the majority of these signals were related to cell proliferation and epithelial growth, including EGFR signaling, FGFR2 signaling, and NRG1 signaling (Fig. S4E). In addition, COL17A1 signaling, which was associated with the maintenance of skin stem cells and had a geroprotective effect (Liu et al., 2019), was also reduced during aging (Fig. S4E). These results suggest that aberrant cell communication may contribute to the decreased proliferative ability of aged gingival epithelia and constitute an important basis for gingival aging.

Next, we performed a joint comparative analysis between the ca-DEGs and annotated hotspot genes from various aging-related diseases known to affect the gingiva, including periodontitis, gingivitis, oral potential malignant disorders [oral leukoplakia (OLK), oral lichen planus (OLP)], and oral squamous cell carcinoma (OSCC). These disease-associated genes were obtained from the DisGeNET platform (Pinero et al., 2017, 2020), and then overlapped with the ca-DEGs. Strikingly, the ca-DEGs were strongly associated with all of these diseases, and in particular, periodontitis (Fig. 4I). Collectively, these findings deciphered the cellular and molecular programs underlying primate gingival aging and suggest implications for human aging-related diseases.

### Reduced YAP activity was identified in the aging process of primate gingival epithelium

**Figure 5.**
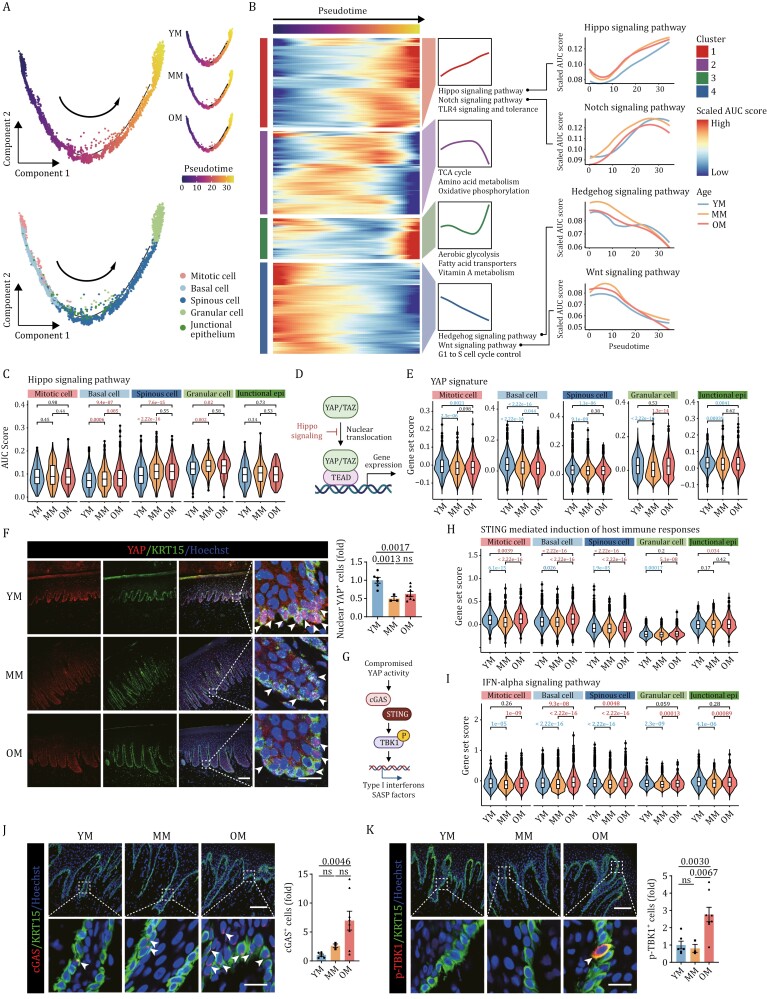
YAP activity declines during aging in primate gingival epithelium. (A) Pseudotime analysis of epithelial cells along the differentiation process in monkey gingiva. Upper, pseudotime scores of epithelial cells. Lower, the distribution of different epithelial cell types along the pseudotime trajectory. YM, young male; MM, middle-aged male; OM, old male. (B) Left: heatmaps showing the scaled activity of pathways along the pseudotime trajectory, which were divided into 4 clusters. Middle: curve charts showing the expression patterns of the specified clusters, with typical pathways indicated. Right: gene set score analyses of the specific pathways in different age groups along the pseudotime trajectory. (C) Gene set score analysis of the Hippo signaling pathway in different cell types during aging. The *P*-values are indicated in the graphs, with the red letter representing a significant upregulation during aging and the black letter representing not significant. (D) Schematic diagram of the Hippo-YAP signaling pathway. (E) Gene set score analysis of keratinocyte YAP signature in different epithelial cells during aging. The *P*-values are indicated in the graphs, with the blue letter representing a significant downregulation during aging, the red letter representing a significant upregulation, and the black letter representing not significant. (F) Immunofluorescence (IF) staining of YAP and KRT15 in gingival tissues of male monkeys. Representative images are shown on the left and quantitative data are shown as means ± SEM on the right. The white arrows indicate nuclear YAP-positive cells. The *P*-values are indicated in the graphs and ns indicates not significant. YM: *n* = 6; MM: *n* = 3; OM: *n* = 8. Scale bars, 100 μm and 20 μm (zoomed-in images). (G) Schematic diagram of the association between the decline of YAP activity and innate immune response. (H and I) Gene set score analysis of STING (H) and IFN-alpha (I) in different epithelial cells during aging. The *P*-values are indicated in the graphs, with the blue letter representing a significant downregulation during aging, the red letter representing a significant upregulation, and the black letter representing not significant. (J) IF staining of cGAS and KRT15 in gingival tissues of male monkeys. Representative images are shown on the left and quantitative data are shown as means ± SEM on the right. The white arrows indicate cGAS-positive cells. The *P*-values are indicated in the graphs and ns indicates not significant. YM: *n* = 6; MM: *n* = 3; OM: *n* = 8. Scale bars, 100 μm and 20 μm (zoomed-in images). (K) IF staining of p-TBK1 and KRT15 in gingival tissues of male monkeys. Representative images are shown on the left and quantitative data are shown as means ± SEM on the right. The white arrows indicate p-TBK1-positive cells. The *P*-values are indicated in the graphs and ns indicates not significant. YM: *n* = 6; MM: *n* = 3; OM: *n* = 8. Scale bars, 100 μm and 20 μm (zoomed-in images).

As epithelial cells appear to be the cell type most susceptible to aging in the gingiva, we focused our next studies on its epithelial cells. Through self-renewal and division of epithelial stem cells residing in the basal layer, the gingival epithelium regenerates continuously throughout life to replace the cells shed from the surface (Calenic et al., 2015). Although the self-renewal capacity of gingival epithelium is generally assumed to be affected by aging, the precise mechanisms remain poorly understood. By performing pseudotime analysis, we simulated the differentiation trajectory of epithelial cells, from mitotic cells, basal cells, and spinous cells, to granular cells (Fig. 5A). Globally, no obvious differences were observed in the cell distribution along the trajectories between different age groups (Fig. 5A). Next, we established the pseudo-time of underlying molecular cascades, in which we identified four patterns of pathway dynamics along the differentiation trajectory (Fig. 5B). Among these, cluster 1 defined pathways that were progressively upregulated with the trajectory, such as the Hippo signaling and the Notch signaling pathway, suggesting that these promote differentiation and are negatively correlated with self-renewal capacity. In contrast, cluster 4 defined pathways that were gradually downregulated along the trajectory, such as the Hedgehog and Wnt signaling pathway, suggesting that these are involved in maintaining self-renewal. Finally, Clusters 2 and 3 showed fluctuating changes along the differentiation trajectory that were mainly related to metabolic pathways.

Next, we compared pathways associated with self-renewal between different age groups. Strikingly, along the trajectory, we found that the level of Hippo signaling was persistently higher in the MM and OM groups relative to the YM group, while the other pathways were not obviously different between different age groups (Fig. 5B). Further, upon comparing gene set scores in different cell types, we discovered that Hippo signaling was upregulated during aging in basal cells, spinous cells, and granular cells (Fig. 5C). YAP is the main effector molecule of Hippo signaling pathway, and upregulation of Hippo signaling can prevent YAP to translocate to the nucleus, and thereby prevent transcription of its downstream target genes (Fu et al., 2022) (Fig. 5D). Consistently, when YAP activity was assessed based on the signature of YAP-targeted genes in keratinocytes (Yuan et al., 2020), the data showed that YAP activity declined distinctly during aging (Fig. 5E). Moreover, when analyzing other YAP-conserved signatures (Cordenonsi et al., 2011; Wang et al., 2018), we found that YAP activity was mainly compromised in basal cells (Fig. S5A and S5B).

Next, we analyzed YAP-expression through IF staining. We found that nuclear YAP was mainly present in KRT15^+^ basal cells and that the proportion of nuclear YAP-positive cells markedly decreased during aging (Figs. 5F and S5C). In addition, we also detected the mRNA levels of *YAP* among different age groups, and the results consistently revealed a decreased mRNA level of *YAP* in the old group, especially in mitotic cells and basal cells (Fig. S5D). Recently, decreased YAP-function was reported to be associated with activation of innate immunity, which led to accelerated aging in stromal cells (Sladitschek-Martens et al., 2022) (Fig. 5G). Consistently, we found that the scores of innate immunity-related gene sets, stimulator of interferon genes (STING), and interferon-alpha (IFN-alpha), were upregulated with age in epithelial cells (Fig. 5H and 5I). The IF analysis confirmed that the expressions of innate immunity-related proteins, cyclic guanosine monophosphate-adenosine monophosphate synthase (cGAS) and phosphorylation of TANK-binding kinase 1 (p-TBK1), significantly increased during aging, and that the positive signals were mainly located in basal cells (Fig. 5J and 5K). Consistently, gene set scores of senescence-associated secretory phenotype (SASP) and inflammatory response were elevated with age in basal cells (Fig. S5E). Taken together, these results indicate that reduced YAP activity may impair the self-renewal and homeostasis of the epithelium in ways that contribute to primate gingival aging.

### Knockdown of *YAP* recapitulated senescent phenotypes in human primary gingival keratinocytes

**Figure 6.**
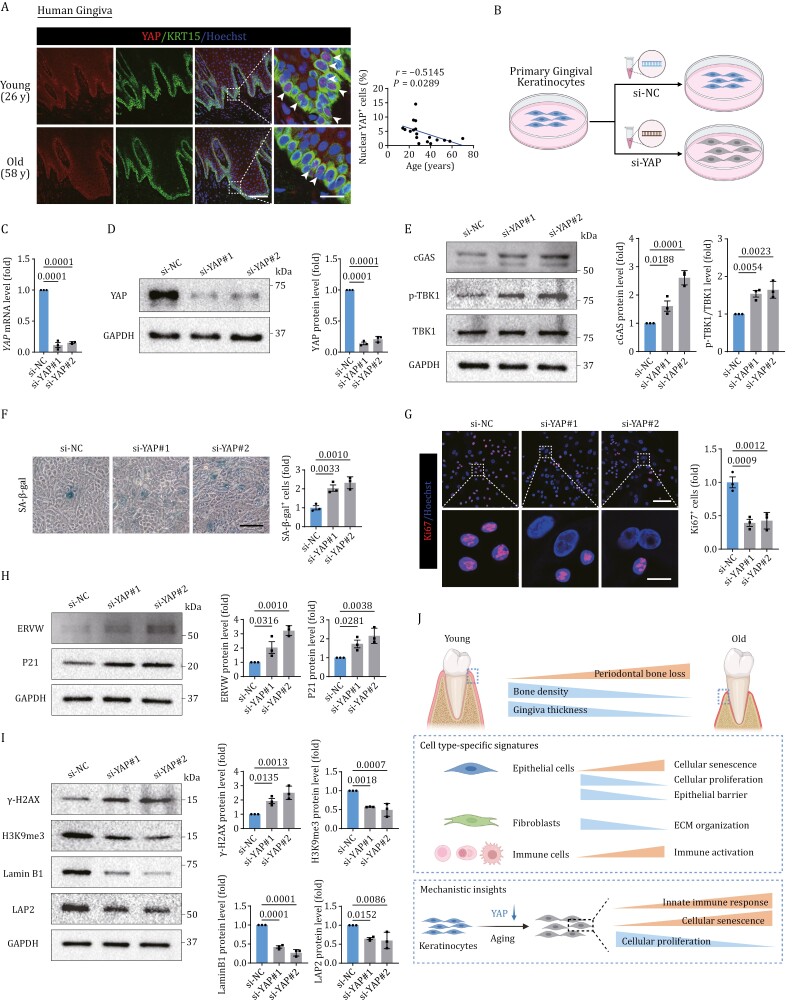
YAP downregulation induced senescent phenotypes in human primary gingival keratinocytes. (A) Immunofluorescence staining of YAP and KRT15 in human healthy gingival tissues. Left, representative images are shown and the white arrows indicate nuclear YAP-positive cells. Right, Pearson regression analysis showing a negative correlation between the proportion of nuclear YAP-positive cells and age. *n* = 18 donors. Scale bars, 100 μm and 20 μm (zoomed-in images). (B) Schematic diagram showing the procedure of *YAP*-knockdown assay in human primary gingival keratinocytes. (C) Validation of the knockdown efficiency by RT-qPCR. *YAP* mRNA levels are quantified as fold changes (si-YAP vs. si-NC) and shown as means ± SEM. The *P*-values are indicated in the graphs. *n* = 3 independent replicates. (D) Validation of the knockdown efficiency by Western blot. YAP protein levels are quantified as fold changes (si-YAP vs. si-NC) and shown as means ± SEM on the right. The *P*-values are indicated in the graphs. *n* = 3 independent replicates. (E) Western blot analysis of cGAS, p-TBK1, and TBK1 protein expression in human primary gingival keratinocytes upon knockdown of *YAP*. Relative protein levels are quantified as fold changes (si-YAP vs. si-NC) and shown as means ± SEM on the right. The *P*-values are indicated in the graphs. *n* = 3 independent replicates. (F) SA-β-gal staining of human primary gingival keratinocytes upon knockdown of *YAP*. Representative images are shown on the left. SA-β-gal positive cells are quantified as fold changes (si-YAP vs. si-NC) and shown as means ± SEM on the right. The *P*-values are indicated in the graphs. *n* = 3 independent replicates. Scale bars, 100 μm. (G) Immunofluorescence staining of Ki67 in human primary gingival keratinocytes upon knockdown of *YAP*. Representative images are shown on the left. Ki67 positive cells are quantified as fold changes (si-YAP vs. si-NC) and shown as means ± SEM on the right. The *P*-values are indicated in the graphs. *n* = 3 independent replicates. Scale bars, 100 μm and 20 μm (zoomed-in images). (H) Western blot analysis of ERVW and P21 protein expression in human primary gingival keratinocytes upon knockdown of *YAP*. Relative protein levels are quantified as fold changes (si-YAP vs. si-NC) and shown as means ± SEM on the right. The *P*-values are indicated in the graphs. *n* = 3 independent replicates. (I) Western blot analysis of γ-H2AX, H3K9me3, Lamin B1, and LAP2 protein expression in human primary gingival keratinocytes upon knockdown of *YAP*. Relative protein levels are quantified as fold changes (si-YAP vs. si-NC) and shown as means ± SEM on the right. The *P*-values are indicated in the graphs. *n* = 3 independent replicates. (J) A schematic illustration showing the phenotypic and transcriptomic signatures of primate gingival aging.

Importantly, in human healthy gingival tissues, YAP was also mainly located in basal cells and markedly declined during aging (Figs. 6A and S6A), in line with results observed in monkey gingival tissues. To tease out the role of YAP in gingival aging, we knocked down *YAP* using siRNA in human primary gingival keratinocytes (Fig. 6B). After siRNA transfection, we used RT-qPCR and western blot analysis to confirm YAP reduction at both RNA and protein expression levels (Fig. 6C and 6D). As expected, *YAP* knockdown resulted in upregulation of innate immunity markers cGAS and p-TBK1 (Fig. 6E). Also, the proportion of SA-β-gal-positive cells was upregulated (Fig. 6F) while the positive rate of Ki67 was downregulated (Fig. 6G). In addition, *YAP* knockdown resulted in elevated expression of senescent markers ERVW and P21 (Fig. 6H), consistent with our observations in the aged primate gingiva (Fig. 2B and 2F). In addition, core features of the genomic and epigenomic instability in aged primate gingiva (Figs. 2D, 2E, S2B and S2C) were replicated in the *YAP*-knockdown cells, manifested by upregulation of γ-H2AX and downregulation of H3K9me3, Lamin B1, and LAP2 (Fig. 6I). In contrast, YAP overexpression in gingival keratinocytes showed rejuvenation effects, demonstrated by a decrease in the positive rate of SA-β-gal and decreased expressions of senescent markers P21 and ERVW (Fig. S6B–E). Altogether, our *in vitro* results indicate that downregulation of YAP recapitulates major phenotypic defects present in the aged monkey gingiva while overexpression of YAP shows rejuvenation effects, highlighting the crucial geroprotective role of YAP in gingival aging.

## Discussion

Gingival recession and exposure of the dental root surface increases with age, and is associated with gingivitis and periodontitis, conditions that affects the vast majority of the elderly. The etiology of gingival recession involves environmental, anatomical and iatrogenic factors (Zucchelli and Mounssif, 2015). In addition, complex biological processes such as antigen presentation (Gonzalez et al., 2014), apoptosis (Gonzalez et al., 2011), circadian rhythm (Ebersole and Gonzalez, 2022), and bone biology (Pandruvada et al., 2016) have been reported to be involved in gingival aging. However, previous studies mainly explored molecular alterations of gingival aging at the bulk level, providing a view of average gene expression of cells and tissues. Because the gingiva is a heterogeneous tissue made up of multiple cell types; bulk level analysis, which does not yield information about specific cell types, falls short in terms of helping us understand the cellular basis of gingival aging. To this end, we systematically analyzed the phenotypic characteristics of gingival aging in primates and established, for the first time, a single-nucleus transcriptomic atlas. These efforts led to the identification of a geroprotective role for YAP in gingival aging, thereby informing the development of potential strategies to intervene in aging-related gingival disorders (Fig. 6J).

Based on our dataset, we identified that aging had the largest impact on epithelial cells. As a vital part of the oral mucosal barrier, epithelial cells participate in immune regulation and form a physical barrier that protects the tissue from external stimuli (Moutsopoulos and Konkel, 2018). In the aging gingiva, we found that epithelial cells, and especially basal cells, were more susceptible to aging than other cell types. This vulnerability manifested as a increase in a series of aging-related indicators, with downstream effects on epithelial proliferation, self-renewal, and barrier function, thereby increasing susceptibility to aging-related diseases. We also revealed other cell type-specific aging-related changes, such as a decline of collagen synthesis and assembly capacity in fibroblasts, activation of a variety of immune cells, and decreased osteogenesis concomitant with increased osteoclastic effect. In summary, our work establishes a robust single-nucleus transcriptome resource that allows us to thoroughly delineate the intricate cell-type-specific regulatory mechanism of primate gingival aging.

Moreover, we identified and validated *in vitro* that the decline of YAP drives the aging of gingival epithelium. YAP, as a mechanotransducer, responds to biomechanical signals and plays a vital role in biological processes such as stem cell replenishment (Driskill and Pan, 2023), organ regeneration (Moya and Halder, 2019), inflammation, fibrosis, and cancer (Panciera et al., 2017). Previous works by us and others revealed that YAP activity alleviated senescence of mesenchymal stem cells (Fu et al., 2019) and stromal cells (Sladitschek-Martens et al., 2022). In contrast, YAP was also reported to accelerate vascular senescence (Pan et al., 2021) and promote the survival of senescent cells (Anerillas et al., 2023), consistent with a multi-faceted and context-dependent role for YAP signaling. To date, the relationship between YAP and gingival aging has not been investigated. The present study, for the first time, reports that decreased YAP activity drives epithelial senescence and plays a critical role in gingival aging. The gingival epithelium is pulled and stabilized around the teeth by mechanical forces exerted by collagen fibers in the lamina propria (Bartold et al., 2000). Thus, aging-associated atrophy of the lamina propria and thinning of collagen fiber bundles would be expected to lead to a decline in mechanical forces on the epithelial cells, and concomitant decreased YAP activity, but this proposed mechanism would need to be explored and confirmed in future work. Here, we validated that YAP knockdown in gingival epithelial cells increased numbers of senescent cells, along with genomic and epigenomic instability, ERV upregulation, and innate immune activation. As all of these phenotypes are powerful drivers of aging (Liu et al., 2023), our *in vitro* evidence highlights the geroprotective role of YAP in gingival aging.

In conclusion, we constructed a comprehensive single-nucleus transcriptomic landscape of primate gingival aging and identified *in vitro* YAP as a geroprotector in primate gingiva. Our work provides an in-depth understanding of gingival aging and serves as a rich resource for developing novel strategies to combat aging-associated gingival diseases, with the ultimate goal of advancing periodontal health and promoting healthy aging.

## Materials and methods

### Animals

The cynomolgus monkeys used in this study were 12 young monkeys (4–5 years old), 6 middle-aged monkeys (10–12 years old), and 18 old monkeys (16–19 years old). The monkeys were raised at the facility in Xieerxin Biology Resource (a certified primate research center in Beijing) at 25°C with a 12-hour light and dark cycle. All animals used in this study were confirmed in advance to have no clinical or experimental history that could potentially affect the physiological aging process. The detailed information of the monkeys is shown in Fig. S1A.

### Tissue sampling

After being completely anesthetized, the cynomolgus monkeys were perfused with phosphate buffers. The full layer of buccal gingival tissue of each site was removed from the maxilla and mandible. After being washed with pre-cooled phosphate buffer saline (PBS), the removed gingival tissues were fixed with 4% paraformaldehyde (PFA) and then paraffin-embedded, or directly embedded with optimal cutting temperature compound (OCT), or frozen in liquid nitrogen for subsequent use. The maxilla and mandible were removed and stored in 4% PFA for subsequent micro-CT analysis. Human healthy gingival samples were obtained during tooth extractions. Inclusion criteria were: no signs/symptoms of gingival inflammation, no bleeding on probing, and a probing depth <3 mm (Caetano et al., 2021; Williams et al., 2021). The collected human gingival tissues were fixed with 4% PFA and then paraffin-embedded. The detailed information of the human donors is shown in Fig. S6A.

### Micro-CT analysis

The maxilla and mandible samples were scanned using the Micro-CT Scanner (PE Quantum FX, USA). The images were reconstructed in three-dimensional form. The measurement of the distance between the cementoenamel junction and alveolar bone crest (CEJ-ABC distance) was performed as described previously with a slight modification (Dutzan et al., 2017; Eskan et al., 2012). Briefly, the CEJ-ABC distances of the premolars and molars of the maxilla and mandible of each monkey were measured and combined to give an average. Bone density was measured at the site of the bone surrounding the mesiobuccal root of the left maxillary first molar, as described previously (Zang et al., 2020).

### Hematoxylin and eosin (H&E) staining

H&E staining was performed as previously described (Zou et al., 2021). Paraffin-embedded gingival sections with a 5 μm thickness were deparaffinized in xylene, rehydrated in a series of gradient alcohols (100%, 100%, 95%, 85%, 75%, 50%), and washed in distilled water. The rehydrated slices were incubated with hematoxylin (Servicebio, China), then washed with tap water to remove excess dye, differentiated in 1% hydrochloric alcohol for 30 s, and washed with tap water for 1 min. The slices were then stained with eosin, dehydrated in a series of gradient alcohols (85%, 95%, 100%, 100%), rendered transparent in xylene, and mounted with a resinous mounting medium. Images were taken with Versa 200 (Leica, Germany).

### Masson’s trichrome staining

Masson’s trichrome staining was performed using the Modified Masson’s Trichrome Staining Kit (Solarbio, G1346) following the manufacturers’ protocol. In brief, the paraffin-embedded gingival sections with a 5 μm thickness were deparaffinized in xylene, rehydrated in a series of gradient alcohols (100%, 100%, 95%, 85%, 75%, 50%), and washed in distilled water. The sections were incubated with Mordant Solution at 60°C for 1 h and washed with running water for 10 min. Then, the sections were incubated sequentially in Celestite Blue Solution for 3 min, in Mayer Hematoxylin Solution for 3 min, in acid differentiation solution for several seconds, and in Ponceau-Acid Fuchsin Solution for 10 min. Between each step, the sections were washed with distilled water. Then, the sections were stained with phosphomolybdic acid solution for 10 min and transferred directly to aniline blue solution for 5 min without washing. The sections were then rinsed in acetic acid solution for 2 min. Finally, the sections were quickly dehydrated with 95% ethanol and absolute ethanol, rendered transparent in xylene, and mounted with a resinous mounting medium. Images were taken with Versa 200 (Leica, Germany).

### Senescence-associated β-galactosidase (SA-β-gal) staining

SA-β-gal staining was performed according to previously published protocol (Huang et al., 2023; Ma et al., 2020, 2021; Wang et al., 2023a). For tissues, the OCT-embedded gingival tissues were cryo-sectioned at a thickness of 20 μm with a Leica CM3050S cryomicrotome, mounted on Superfrost Plus microslides (VWR), and stored at −80°C until use. The sections were thawed at room temperature and washed with PBS, fixed in fixation buffer (2% formaldehyde and 0.2% glutaraldehyde) at room temperature for 5 min, washed with PBS twice, and stained with freshly prepared staining solution (5 mmol/L K_4_[Fe(CN)_6_], 5 mmol/L K_3_[Fe(CN)_6_], 150 mmol/L NaCl, 2 mmol/L MgCl_2_, 40 mmol/L citric acid/Na phosphate buffer, 1 mg/mL X-gal) at 37°C. X-gal was purchased from Amresco and all the other reagents were from Sigma-Aldrich. Continue to observe until the desired degree of staining was achieved. The sections were then counterstained with nuclear fast red, dehydrated in gradient alcohols, rendered transparent in xylene, and mounted with a resinous mounting medium. For cells, the cultured cells were washed with PBS, fixed in fixation buffer at room temperature for 5 min, washed with PBS twice, and stained with freshly prepared staining solution at 37°C overnight. Images were taken with a Nikon microscope imaging system, and the percentages of SA-β-gal-positive cells were quantified with ImageJ.

### Immunohistochemistry (IHC) staining

IHC staining was performed as previously described (Huang et al., 2023; Jing et al., 2023). Briefly, the paraffin-embedded gingival sections with a 5 μm thickness were deparaffinized in xylene, rehydrated in a series of gradient alcohols (100%, 100%, 95%, 85%, 75%, 50%), and washed in distilled water. Antigen retrieval was performed in citrate buffer (pH 6.0), after which sections were microwaved for 25 min, and cooled to room temperature. The sections were rinsed three times with PBS and then permeabilized with 0.4% Triton X-100 in PBS for 1 h at room temperature. After that, the sections were incubated with 3% H_2_O_2_ for 10 min at room temperature to inactivate endogenous peroxidase. Sections were then incubated with primary antibodies (dilution with 5% donkey serum in PBS) at 4°C overnight. The next day, the sections were rinsed with PBS three times and incubated with HRP-conjugated secondary antibodies (ZSGB-BIO) for 1 h at room temperature. The signal was visualized using a DAB Staining Kit (ZSGB-BIO) and counterstained with hematoxylin. Finally, the sections were dehydrated in gradient alcohols, rendered transparent in xylene, and mounted with a resinous mounting medium. Images were taken with Versa 200 (Leica, Germany). The primary antibodies used in this study for IHC staining are listed as follows: P21 (CST, 2947S, 1:200), P-H3 (CST, 3377S, 1:500), PCNA (Abcam, ab29, 1:6,000), γ-H2AX (CST, 9718S, 1:400), LAP2 (BD, 611000, 1:100), ERVW (Abcam, ab179693, 1:400). The IHC staining results were quantified by calculating the proportions of positive cells using ImageJ.

### Immunofluorescence (IF) staining

IF staining was performed as previously described (Huang et al., 2023; Wang et al., 2020; Yang et al., 2023). For the OCT-embedded gingival tissues, the sections with a 10 μm thickness were washed with PBS, fixed with 4% PFA for 20 min at 4°C, permeabilized with 0.4% Triton X-100 in PBS for 30 min at room temperature, and incubated with blocking buffer (10% donkey serum in PBS) for 1 h at room temperature. After that, the sections were incubated with primary antibodies (dilution with 5% donkey serum in PBS) at 4°C overnight, followed by incubation with fluorescence-labeled secondary antibodies (Thermo Fisher, 1:500) and Hoechst 33342 (Thermo Fisher, H3570, 1:1000) for 1 h at room temperature in the dark. Then, the sections were mounted with a VECTERSHIELD anti-fading mounting medium (Vector Laboratories, H-1000). Images were obtained using a confocal laser scanning microscope (Nikon).

For the paraffin-embedded gingival tissues, the sections with a 5 μm thickness were routinely deparaffinized and rehydrated. Antigen retrieval was performed in citrate buffer (pH 6.0), after which the sections were microwaved for 25 min, and cooled to room temperature. The sections were permeabilized with 0.4% Triton X-100 in PBS for 1 h at room temperature. Then, the sections were incubated with a blocking buffer (10% donkey serum in PBS) for 1 h at room temperature. The next steps are the same as for OCT-embedded samples.

For cells, microscope coverslips with seeded cells were washed with PBS, fixed with 4% PFA for 15 min, and permeabilized with 0.4% Triton X-100 in PBS for 30 min at room temperature. Then, the coverslips were incubated with blocking buffer (10% donkey serum in PBS) for 1 h at room temperature. The next steps are the same as for tissue samples.

The primary antibodies used in this study for IF staining are listed as follows: H3K9me3 (Abcam, ab8898, 1:400), Lamin B1 (Abcam, ab16048, 1:400), CD45 (Abcam, ab10558, 1:300), CD79B (CST, 96024, 1:250), IGKC (Proteintech, 14678-1-AP, 1:100), CD163 (Abcam, ab182422, 1:200), E-cadherin (R&D, AF748, 1:50), CX-43 (Sigma, C6219, 1:600), YAP (CST, 14074, 1:200), KRT15 (Invitrogen, MA5-11344, 1:200), cGAS (CST, 79978, 1:200), p-TBK1 (CST, 5483, 1:100), and Ki67 (Abcam, ab15580, 1:500). The IF staining results were quantified by calculating the proportion of positive cells or the average immunofluorescence intensity using ImageJ.

### SA-β-gal and IF co-staining

SA-β-gal and IF co-staining was performed as previously described (Huang et al., 2023; Zhang et al., 2023b). Briefly, after the SA-β-gal staining procedure, the sections were washed with PBS, fixed with 4% PFA for 20 min, and permeabilized with 0.4% Triton X-100 in PBS for 1 h at room temperature. The sections were incubated with blocking buffer (10% donkey serum in PBS) for 1 h at room temperature, then followed the same steps as IF staining. Images were obtained using a confocal laser scanning microscope (Nikon) and further transformed and merged using ImageJ.

### Cell culture

The human primary gingival keratinocytes (PCS-200-014) were obtained from ATCC (USA). The cells were cultured in KGM-2 Keratinocyte Growth Medium-2 BulletKit (LONZA, cc-3107) supplemented with 0.5 ng/mL human recombinant TGF-alpha (STEMCELL, #78123) and 6 mmol/L GlutaMAX (GIBCO, 35050-061).

### Knockdown of *YAP* using small interfering RNA (siRNA)

siRNA-mediated knockdown of *YAP* in human primary gingival keratinocytes was performed as previously described (Huang et al., 2023; Jing et al., 2023). siRNAs were synthesized by Tsingke Biotechnology (China). Lipofectamine® RNAiMAX Reagent (Invitrogen, 13778-150) was used for the transfections of RNA oligonucleotides according to the manufacturer’s instructions. Cells were collected 48–72 h after transfection for subsequent analysis. The sequence information is listed in Table S1.

### Plasmid construction and lentivirus packaging

For the overexpression strategy, YAP cDNA was generated by PCR amplification and then cloned into the pLE4 vector (a kind gift from Dr. Tomoaki Hishida) that had been pre-cleaved by *BamH1* and *MluI*. The pLE4 vector expressing luciferase (Luc) was used as control. HEK293T cells were transfected with lentiviral overexpression plasmid and lentiviral packaging vectors psPAX2 (Addgene #12260) and pMD2.G (Addgene #12259). The viral particles were then collected at 48 and 72 h post-transfection, respectively, and concentrated by ultracentrifugation at 19,400 ×*g* for 2.5 h. The primer information is listed in Table S1.

### Quantitative reverse transcription PCR (RT-qPCR)

Total RNAs of human primary gingival keratinocytes were extracted using Trizol (Invitrogen, 15596018) according to the manufacturer’s instructions. The cDNAs were synthesized using the HiScript III RT SuperMix for qPCR (+ gDNA wiper) (Vazyme, R323-01). RT-qPCR was conducted with THUNDERBIRD SYBR® qPCR Mix (TOYOBO, QPS-201C) on Bio-Rad CFX Opus 384 Real-Time PCR System. The RT-qPCR primers used in this study are listed in Table S1.

### Western blot (WB) analysis

WB was performed as described previously (Jing et al., 2023; Zhang et al., 2023b). Briefly, cell pellets were lysed in RIPA lysate buffer (Beyotime, P0013B) supplemented with protease inhibitor (Roche, 4693159001) and phosphatase inhibitor (Roche, 4906837001). The protein lysates were quantified with a BCA kit (Dingguo biotechnology, BCA02) and boiled in SDS-loading buffer for 10 min. The samples were subjected to SDS-PAGE and subsequently transferred to a PVDF membrane (Merck Millipore). The membranes were then blocked with 5% non-fat milk and incubated with primary antibodies at 4°C overnight. After washed with TBST, the membranes were incubated with HRP-conjugated secondary antibodies for 1 h at room temperature. The signals were captured using the ChemiDoc XRS system (Bio-Rad) and quantified with Image J. The antibodies used in this study for WB are listed as follows: YAP (CST, 14074, 1:1000), γ-H2AX (CST, 9718S, 1:1000), H3K9me3 (Abcam, ab8898, 1:3,000), Lamin B1 (Abcam, ab16048, 1:2,000), LAP2 (BD, 611000, 1:500), ERVW (ImmunoWay, YN5333, 1:1000), cGAS (CST, 79978, 1:1000), p-TBK1 (CST, 5483, 1:1000), TBK1 (CST, 3504, 1:1000), P21 (CST, 2947S, 1:1000), GAPDH (Santa Cruz, sc-365062, 1:1000), goat anti-Rabbit IgG (ZSGB-bio, ZB-2307, 1:5,000), and goat anti-Mouse IgG (ZSGB-bio, ZB-2305, 1:5,000).

### Bulk RNA-seq library construction and sequencing

Total RNAs from individual monkey gingival tissues were extracted using TRIzol. The gingival tissues were taken from the first molars, with no obvious redness and a periodontal probing depth <3 mm. RNA quality control, library construction, and high-throughput sequencing were performed for each sample by Novogene Bioinformatics Technology Co., Ltd. Briefly, sequencing libraries were prepared using NEBNext^®^ Ultra^TM^ RNA Library Prep Kit for Illumina^®^ and individually indexed. The resultant libraries were analyzed on an Illumina paired-end sequencing platform by 150-bp read length.

### Bulk RNA-seq data processing

Raw paired-end reads were trimmed by Trim Galore software (version 0.6.7) to trim adapter sequences and remove low-quality reads. The cleaned reads were mapped to the cynomolgus monkey (*Macaca fascicularis*) MacFas5.0 using STAR (version 2.7.1a) (Dobin et al., 2013). Read counts for each gene were calculated by featureCounts (version 2.0.1) (Liao et al., 2014). Differentially expressed genes (DEGs) between different age samples were calculated by R package DESeq2 (version 1.34.0) (Love et al., 2014) with the cutoff values of Benjamini-Hochberg adjusted *P*-value < 0.05 and |log_2_(fold change)| > 0.5. DEGs are listed in Table S2.

### Nuclei isolation and snRNA-seq on the 10× genomics platform

Nuclei isolation was performed following a previously published protocol with minor modifications (Krishnaswami et al., 2016). In brief, a piece of frozen monkey gingival tissues (taken from the first molars, with no obvious redness and a periodontal probing depth <3 mm; YM: *n* = 4, MM: *n* = 3, OM: *n* = 4) was ground into powder separately with liquid nitrogen. The powder was then homogenized in 1.0 mL homogenization buffer (250 mmol/L sucrose, 25 mmol/L KCl, 5 mmol/L MgCl_2_, 10 mmol/L Tris buffer [pH 8.0], 1 μmol/L DTT, 1× protease inhibitor, 0.4 U/μL RNaseIn, 0.2 U/μL Superasin, 0.1% Triton X-100, 1 μmol/L propidium iodide [PI], and 10 ng/mL Hoechst 33342 in Nuclease-free water) using a freezing multi-sample tissue grinding system (60 Hz for 60 s, 3 times). The homogenized samples were filtered through a 40 μm cell strainer (BD Falcon), centrifuged at 1000 ×*g* for 8 min at 4°C, and resuspended in PBS supplemented with 0.3% BSA. PI and Hoechst 33342 double-positive nuclei were sorted by fluorescence-activated cell sorting (FACS) (BD Influx) and counted with a dual-fluorescence cell counter (Luna-FLTM, Logos Biosystems). Mononuclear capture was conducted with a 10× genomics single-cell 3ʹ system. Approximately 7,000 nuclei were captured for each sample following the standard 10× capture and library construction (10× Genomics) and then sequenced on a NovaSeq 6000 sequencing platform (Illumina, 20012866).

### Processing and quality control of snRNA-seq data

Raw sequencing reads of monkey gingiva were aligned to the transcriptome reference (Ensemble, Macaca fascicularis 5.0) and counted using Cell Ranger (version 6.0.2) with the default parameters. The raw count matrix was filtered using CellBender (version 0.2.0) (Fleming et al., 2023) to eliminate the contamination of background mRNA, and Seurat (version 4.1.0) (Butler et al., 2018) was used to remove cells with fewer than 200 genes or a mitochondrial ratio greater than 10%. DoubletFinder (version 2.0.3) (McGinnis et al., 2019) was used to remove doublet. The nucleus number, mean reads per nucleus, gene number per nucleus, genome mapping rate, unique molecular identifier (UMI) number per nucleus, and percentage of mitochondrial genes across each gingival sample of monkeys are shown in Fig. S3A.

### Integration, clustering, and identification of cell types

The following steps were processed individually for monkey gingiva datasets. First, the count matrix of each sample was intergraded using Seurat reciprocal PCA workflows with the default parameters. After data integration and scaling, dimensional reduction was performed using the “RunPCA” and “RunUMAP” functions with the first 20 principal components, and clustering was performed using “FindNeighbors” and “FindClusters” functions with the resolution of two. The marker genes of each cell type were analyzed using the “FindAllMarkers” function with the Wilcoxon rank-sum test. Clusters with cell number less than 200 cells were filtered before cell types identification.

### Aging-associated differentially expressed genes analysis

Aging-associated differentially expressed genes (aging-associated DEGs) between different age groups in monkey gingiva were analyzed by the function of “FindMarkers” using Wilcoxon Rank Sum test, and were identified with the cutoff of |Log_2_FC| > 0.25 and adjusted *P* values < 0.05. Cell types with less than 500 cells, such as the mast cell, and neuron cell, were not used for downstream analysis. The aging-associated DEGs are listed in Table S3.

### Chronological age differentially expressed genes analysis

To identify chronological age differentially expressed genes (ca-DEGs), we undertook a series of steps. Firstly, aging-associated DEGs between different ages were screened and pooled by cell type. Subsequently, the cells of CPM expression matrix were ordered according to age and the expression was smoothed by loess regression with a smoothing parameter of “span = 1”. Finally, we employed linear regression to identify genes that demonstrated continuous up-regulation and down-regulation patterns. The ca-DEGs are listed in Table S4.

### Cell identity analysis

The cell identity genes for each cell type were calculated using the “FindAllMarkers” function in young cells’ data with a cutoff of adjusted *P*-values < 0.05, Log_2_FC > 1, and pct.1 > 10%. We selected all marker genes to score the corresponding cell types using the “AddModuleScore” function.

### Gene Ontology (GO) enrichment analysis

GO enrichment analysis was performed with Metascape (Zhou et al., 2019). Representative terms were visualized with ggplot2 R package (version 3.3.5) (Wickham, 2016).

### Analysis of transcription factor regulatory network

Transcriptional regulatory network was analyzed by SCENIC (version 1.1.2.2) (Aibar et al., 2017) with default parameters. The reference of transcription factors (TFs) was downloaded from RcisTarget (version 1.14.0) with hg19. Cell types with cell more than 5,000 were subsampled to 5,000 cells, and only ca-DEGs were used as input for transcriptional regulator inferring. Only target genes with high-confidence annotation were selected for the downstream analysis.

### Cell–cell communication analysis

CellPhoneDB software (version 2.1.7) was used to calculate the cell–cell communication for each age using default parameters (Efremova et al., 2020). Only receptors and ligands with expression ratio greater than 10% of each cell type were included in the calculation, and only interaction pairs with *P *< 0.05 were used for downstream analysis.

### Pseudotime analysis

Monocle2 (version 2.22.0) (Qiu et al., 2017) was utilized to reconstruct the developmental trajectory of epithelial cells. The count matrix was used as input, and the “differentialGeneTest” function was employed to identify genes that distinguish cell types, with a *q*-value < 0.1. The “DDRTree” method was then applied to the reduced-dimensional representation of the cells in order to estimate the cellular progression along the pseudo-temporal trajectory.

### Pathway analysis and visualization

The AUCell package was utilized to calculate the pathway activation for each cell based on the Wikipathway gene set and generate the pathway activation matrix. Age-specific and cell-type-specific pathways were independently calculated by employing the “FindAllMarkers” function, applying a threshold of an adjusted *P*-value less than 0.05. Subsequently, their overlapping pathways were used for downstream analysis. Lastly, the pathway activation data was clustered and visualized using the pheatmap package.

### Gene set score analysis

The public gene sets were obtained from the MSigDB database (Liberzon et al., 2015). These gene sets were utilized to calculate scores for each input cell using the Seurat function “AddModuleScore”. The discrepancies in scores between different age samples were analyzed using the Wilcoxon test from the ggpubr R package (version 0.2.4).

### Statistical analyses

All experimental data were statistically analyzed using GraphPad Prism (V8). Results were presented as mean ± SEM. Comparisons were conducted using a one-way ANOVA (parametric) test with post hoc test as appropriate. *P* values lower than 0.05 are considered statistically significant.

## Supplementary data

The online version contains supplementary material available at https://doi.org/10.1093/procel/pwae017.

pwae017_suppl_Supplementary_Materials

## Data Availability

The original sequencing data in this study have been deposited in the Genome Sequence Archive in the National Genomics Data Center, Beijing Institute of Genomics (China National Center for Bioinformation) of the Chinese Academy of Sciences, with accession number CRA014943.

## Code availability

All the custom codes are available from the corresponding authors upon reasonable request.
