# Supplementary material for: Single-nucleus transcriptomics uncovers a geroprotective role of YAP in primate gingival aging
Source: Protein Cell. 2024 Apr 5;15(8):612–32. doi: 10.1093/procel/pwae017 (PMC11259548; doi:10.1093/procel/pwae017)

Supplementary Figure Legends

Figure S1. Histological features of periodontal aging in cynomolgus monkeys.

(A) Information of the monkeys used in this study.

(B-C) Quantitative data of hematoxylin and eosin (H&E) staining of male (B) and female (C) monkey gingival tissues. Data are shown as means ± SEM.

(D-E) Quantitative data of Masson's trichrome staining of male (D) and female (E) monkey gingival tissues. Data are shown as means ± SEM.

YM, young male; MM, middle-aged male; OM, old male; YF, young female; MF, middle-aged female; OF, old female. The *P*-values are indicated in the graphs, and ns indicates not significant.

Figure S2. Accumulation of aging-associated indicators in the aged primate gingiva.

(A) Immunohistochemistry staining of PCNA in gingival tissues of male monkeys. Representative images are shown on the left and quantitative data are shown as means ± SEM on the right. Scale bars, 50 μm.

(B-C) Immunofluorescence staining of H3K9me3 (B) and Lamin B1 (C) in gingival tissues of male monkeys. Representative images are shown on the left and quantitative data are shown as means ± SEM on the right. Scale bars, 100 μm and 20 μm (zoomed-in images).

(D) SA-β-gal staining of female monkey gingival tissues (upper) and counterstaining with nuclear fast red (lower). Representative images are shown on the left and quantitative data are shown as means ± SEM on the right. The yellow arrows indicate the SA-β-gal-positive cells. Scale bars, 200 μm and 10 μm (counterstaining).

(E-J) Immunohistochemistry staining of P21 (E), P-H3 (F), PCNA (G), γ-H2AX (H), LAP2 (I), and ERVW (J) in gingival tissues of female monkeys. Representative images are shown on the left and quantitative data are shown as means ± SEM on the right. The red arrows indicate the positive cells. Scale bars, 50 μm.

YM, young male (N=6); MM, middle-aged male (N=3); OM, old male (N=8); YF, young female (N=6); MF, middle-aged female (N=3); OF, old female (N=10). The *P*-values are indicated in the graphs and ns indicates not significant.

Figure S3. Single-nucleus transcriptomic atlas of the primate gingiva during aging.

(A) Quality control of the single-nucleus RNA sequencing data of monkey gingiva. Plots showing the $\log_{10}$(nucleus number), mean reads per nucleus, genome mapping rate, gene number per nucleus, unique molecular identifier (UMI) number per nucleus, and percentage of mitochondrial genes across each gingival sample of monkeys.

(B) Uniform manifold approximation and projection (UMAP) plots showing the expression profiles

of indicated cell-type-specific marker genes in monkey gingiva.

(C) Dot plots showing the expression levels of representative marker genes for each cell type.

YM, young male; MM, middle-aged male; OM, old male.

Figure S4. Aging-associated cellular and molecular characteristics of the primate gingiva.

(A) Dot plots showing the representative shared GO terms enriched for the upregulated (upper) and downregulated (lower) differentially expressed genes (DEGs) across different cell types in the monkey gingiva between different groups.

(B) Representative cell type-specific GO terms of the age-dependent upregulated (red) and downregulated (blue) DEGs in the monkey gingiva.

(C) Heatmaps showing the cell-cell interaction numbers across cell types in different aged groups of monkey gingiva.

(D) Heatmaps showing the alteration of cell-cell interaction numbers during aging across cell types of monkey gingiva.

(E) Dot plots showing the representative declined ligand-receptor pairs during aging across cell types of monkey gingiva.

YM, young male; MM, middle-aged male; OM, old male; SC, spinous cell; GC, granular cell; JE, junctional epithelium; Mito, mitotic cell; TC, T cell; BC, B cell; Macro, macrophage; EC, endothelial cell; FB, fibroblast; Peri, pericyte; Y, young; M, middle-aged; O, old.

Figure S5. The decline of YAP activity in primate gingival epithelium during aging.

(A-B) Gene set score analysis of conserved YAP signature in different epithelial cells during aging. The *P*-values are indicated in the graphs, with the blue letter representing a significant downregulation during aging, the red letter representing a significant upregulation, and the black letter representing not significant. YM, young male; MM, middle-aged male; OM, old male.

(C) Immunofluorescence staining of YAP and KRT15 in gingival tissues of female monkeys. Representative images are shown on the left and quantitative data are shown as means ± SEM on the right. The white arrows indicate nuclear YAP-positive cells. The *P*-values are indicated in the graphs and ns indicates not significant. YF, young female (N=6); MF, middle-aged female (N=3); OF, old female (N=10). Scale bars, 100 μm and 20 μm (zoomed-in images).

(D) Violin plots showing the mRNA levels of *YAP* in different epithelial cells during aging. The *P*-values are indicated in the graphs, with the blue letter representing a significant downregulation during aging, the red letter representing a significant upregulation, and the black letter representing not significant.

(E) Gene set score analysis of senescence-associated secretory phenotype (SASP) and inflammatory response in basal cells during aging. The *P*-values are indicated in the graphs, with the red letter

representing a significant upregulation during aging.

Figure S6. YAP overexpression showed rejuvenation effects in human primary gingival keratinocytes

(A) The information of the healthy gingival tissue donors.

(B) Schematic diagram showing the procedure of YAP-overexpression assay in human primary gingival keratinocytes.

(C) Validation of the overexpression efficiency by Western blot. YAP protein levels are quantified as fold changes (Flag-YAP vs. Flag-Luc) and shown as means ± SEM on the right. The *P*-value is indicated in the graph. N = 3 independent replicates.

(D) SA-β-gal staining of human primary gingival keratinocytes upon overexpression of YAP. Representative images are shown on the left. SA-β-gal positive cells are quantified as fold changes (Flag-YAP vs. Flag-Luc) and shown as means ± SEM on the right. The *P*-value is indicated in the graph. N = 3 independent replicates. Scale bars, 200 μm.

(E) Western blot analysis of P21 and ERVW protein expression in human primary gingival keratinocytes upon overexpression of YAP. Relative protein levels are quantified as fold changes (Flag-YAP vs. Flag-Luc) and shown as means ± SEM on the right. The *P*-value is indicated in the graph. N = 3 independent replicates.

Supplementary Tables

Table S1. The sequence information.

Table S2. List of DEGs of bulk RNA-seq.

Table S3. List of aging-associated DEGs between different age groups of snRNA-seq.

Table S4. List of ca-DEGs of snRNA-seq.

Figure S1

A

Male			Female		
**Group**	**Sample number**	**Age (Year)**	**Group**	**Sample number**	**Age (Year)**
**Young**	YM-1	5	**Young**	YF-1	4
	YM-2	5		YF-2	4
	YM-3	5		YF-3	4
	YM-4	5		YF-4	4
	YM-5	5		YF-5	4
	YM-6	5		YF-6	4
**Middle-aged**	MM-1	10	**Middle-aged**	MF-1	12
	MM-2	12		MF-2	11
	MM-3	11		MF-3	11
**Old**	OM-1	18	**Old**	OF-1	17
	OM-2	17		OF-2	16
	OM-3	16		OF-3	17
	OM-4	18		OF-4	16
	OM-5	18		OF-5	19
	OM-6	16		OF-6	16
	OM-7	17		OF-7	18
	OM-8	16		OF-8	18
				OF-9	18
				OF-10	17

B C D E

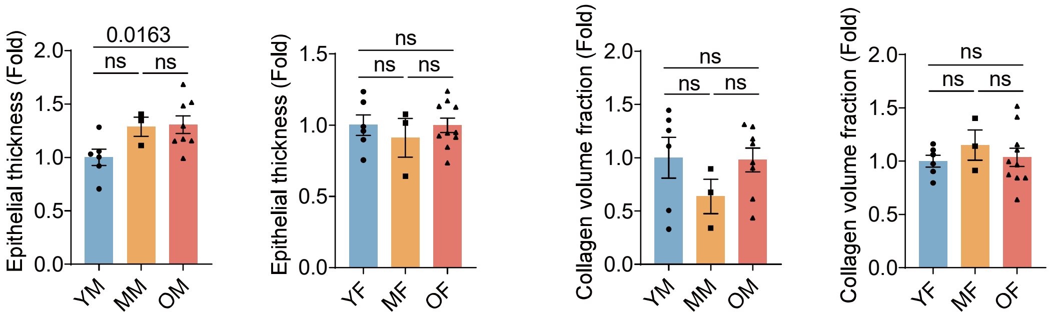

Figure S2

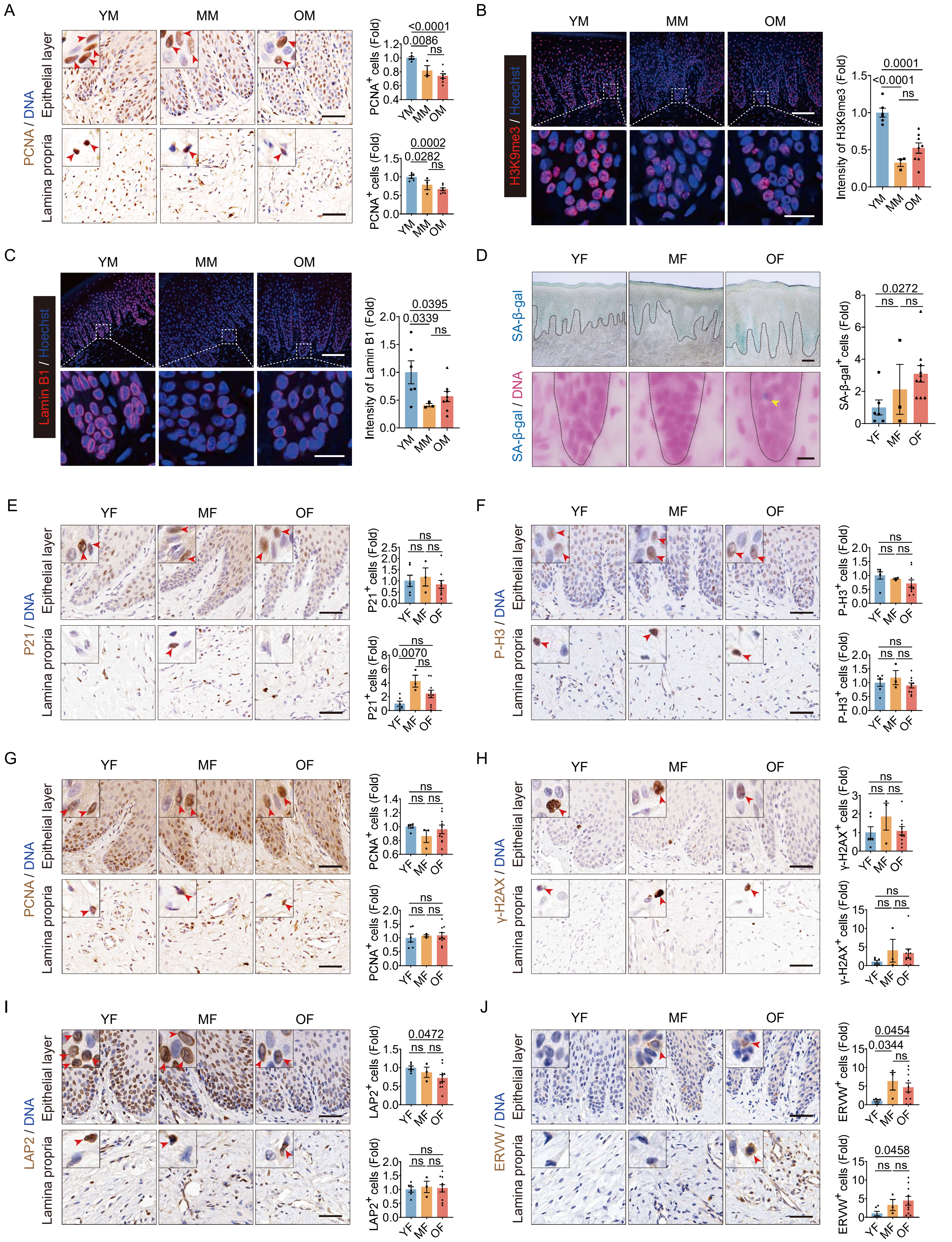
A
YM
MM
OM
PCNA / DNA
Epithelial layer
Lamina propria
PCNA+ cells (Fold)
<0.0001
0.0086
ns
0.0002
0.0282
B
H3K9me3 / Hoechst
Intensity of H3K9me3 (Fold)
0.0001
<0.0001
C
Lamin B1 / Hoechst
Intensity of Lamin B1 (Fold)
0.0395
0.0339
D
YF
MF
OF
SA-β-gal
SA-β-gal / DNA
SA-β-gal+ cells (Fold)
0.0272
E
P21 / DNA
P21+ cells (Fold)
0.0070
F
P-H3 / DNA
P-H3+ cells (Fold)
G
H
γ-H2AX / DNA
γ-H2AX+ cells (Fold)
I
LAP2 / DNA
LAP2+ cells (Fold)
0.0472
J
ERVW / DNA
ERVW+ cells (Fold)
0.0454
0.0344
0.0458

Figure S3

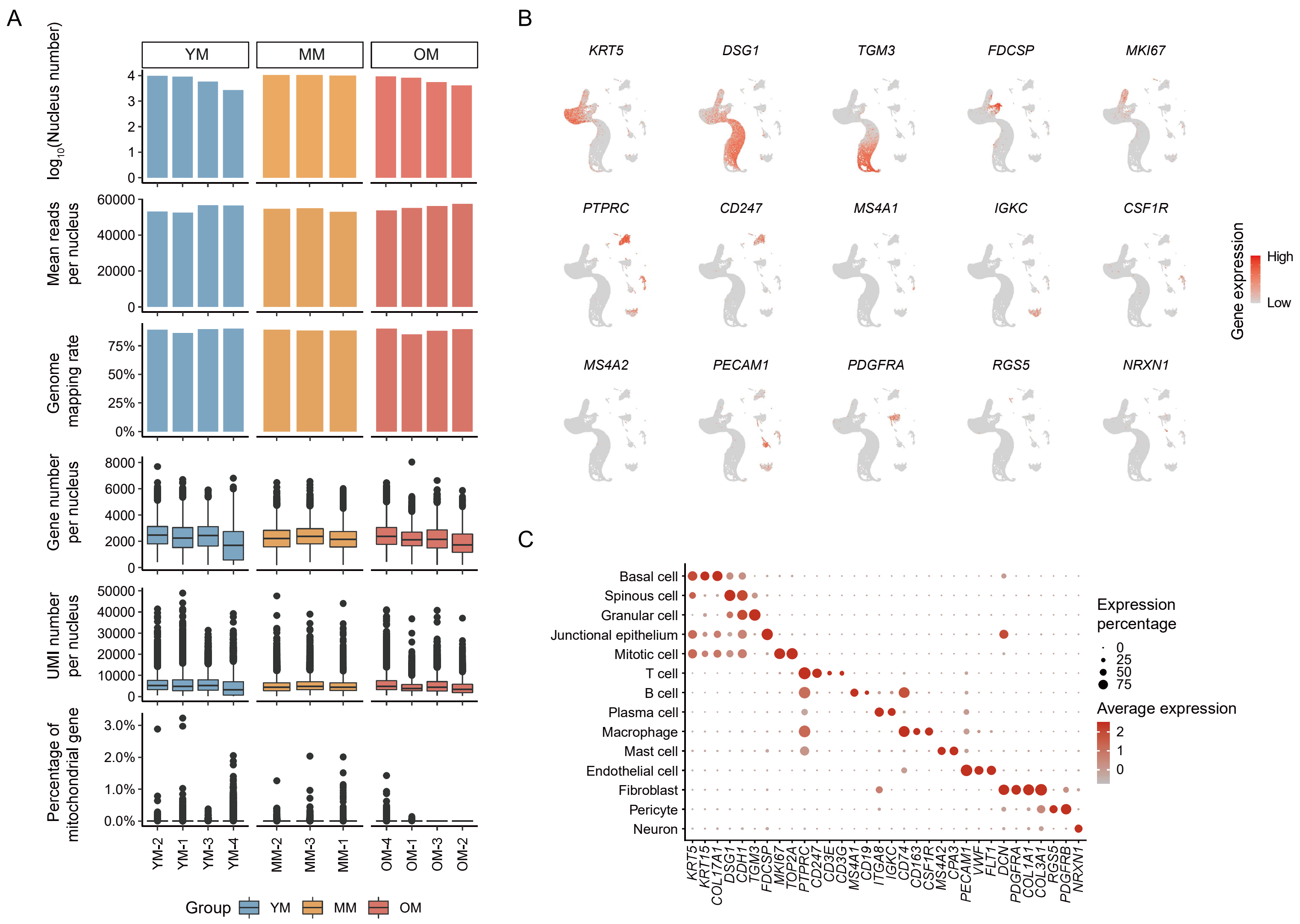

A
YM
MM
OM
log10(Nucleus number)
Mean reads per nucleus
Genome mapping rate
Gene number per nucleus
UMI number per nucleus
Percentage of mitochondrial gene
YM-2
YM-1
YM-3
YM-4
MM-2
MM-3
MM-1
OM-4
OM-1
OM-3
OM-2
Group
YM
MM
OM
B
KRT5
DSG1
TGM3
FDCSP
MKI67
PTPRC
CD247
MS4A1
IGKC
CSF1R
MS4A2
PECAM1
PDGFRA
RGS5
NRXN1
Gene expression
High
Low
C
Basal cell
Spinous cell
Granular cell
Junctional epithelium
Mitotic cell
T cell
B cell
Plasma cell
Macrophage
Mast cell
Endothelial cell
Fibroblast
Pericyte
Neuron
KRT5
KRT15
COL17A1
DSG1
CDH1
TGM3
FDCSP
MKI67
TOP2A
PTPRC
CD247
CD3E
CD3G
MS4A1
CD19
ITGA8
IGKC
CD74
CD163
CSF1R
MS4A2
CPA3
PECAM1
VWF
FLT1
DCN
PDGFRA
COL1A1
COL3A1
RGS5
PDGFRB
NRXN1
Expression percentage
0
25
50
75
Average expression
2
1
0

Figure S4

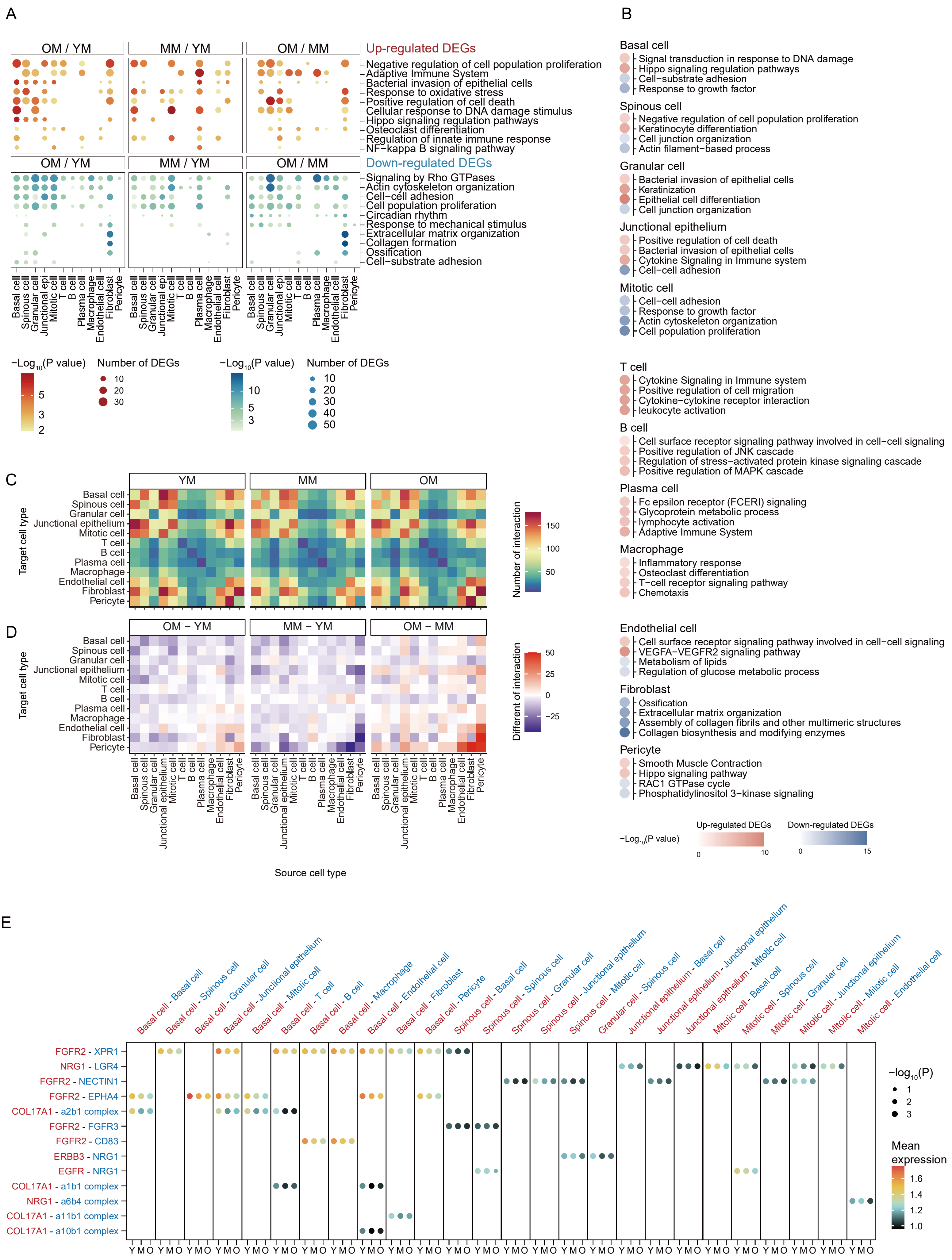
A
OM / YM
MM / YM
OM / MM
Up-regulated DEGs
Negative regulation of cell population proliferation
Adaptive Immune System
Bacterial invasion of epithelial cells
Response to oxidative stress
Positive regulation of cell death
Cellular response to DNA damage stimulus
Hippo signaling regulation pathways
Osteoclast differentiation
Regulation of innate immune response
NF−kappa B signaling pathway
Down-regulated DEGs
Signaling by Rho GTPases
Actin cytoskeleton organization
Cell−cell adhesion
Cell population proliferation
Circadian rhythm
Response to mechanical stimulus
Extracellular matrix organization
Collagen formation
Ossification
Cell−substrate adhesion
Basal cell
Spinous cell
Granular cell
Junctional epi
Mitotic cell
T cell
B cell
Plasma cell
Macrophage
Endothelial cell
Fibroblast
Pericyte
−Log10(P value)
Number of DEGs
B
Basal cell
Signal transduction in response to DNA damage
Hippo signaling regulation pathways
Cell−substrate adhesion
Response to growth factor
Spinous cell
Negative regulation of cell population proliferation
Keratinocyte differentiation
Cell junction organization
Actin filament−based process
Granular cell
Bacterial invasion of epithelial cells
Keratinization
Epithelial cell differentiation
Cell junction organization
Junctional epithelium
Positive regulation of cell death
Bacterial invasion of epithelial cells
Cytokine Signaling in Immune system
Cell−cell adhesion
Mitotic cell
Cell−cell adhesion
Response to growth factor
Actin cytoskeleton organization
Cell population proliferation
T cell
Cytokine Signaling in Immune system
Positive regulation of cell migration
Cytokine−cytokine receptor interaction
leukocyte activation
B cell
Cell surface receptor signaling pathway involved in cell−cell signaling
Positive regulation of JNK cascade
Regulation of stress−activated protein kinase signaling cascade
Positive regulation of MAPK cascade
Plasma cell
Fc epsilon receptor (FCERI) signaling
Glycoprotein metabolic process
lymphocyte activation
Adaptive Immune System
Macrophage
Inflammatory response
Osteoclast differentiation
T−cell receptor signaling pathway
Chemotaxis
Endothelial cell
Cell surface receptor signaling pathway involved in cell−cell signaling
VEGFA−VEGFR2 signaling pathway
Metabolism of lipids
Regulation of glucose metabolic process
Fibroblast
Ossification
Extracellular matrix organization
Assembly of collagen fibrils and other multimeric structures
Collagen biosynthesis and modifying enzymes
Pericyte
Smooth Muscle Contraction
Hippo signaling pathway
RAC1 GTPase cycle
Phosphatidylinositol 3−kinase signaling
−Log10(P value)
Up-regulated DEGs
Down-regulated DEGs
C
YM
MM
OM
Target cell type
Junctional epithelium
Number of interaction
D
OM − YM
MM − YM
OM − MM
Different of interaction
Source cell type
E
FGFR2 - XPR1
NRG1 - LGR4
FGFR2 - NECTIN1
FGFR2 - EPHA4
COL17A1 - a2b1 complex
FGFR2 - FGFR3
FGFR2 - CD83
ERBB3 - NRG1
EGFR - NRG1
COL17A1 - a1b1 complex
NRG1 - a6b4 complex
COL17A1 - a11b1 complex
COL17A1 - a10b1 complex
Basal cell - Basal cell
Basal cell - Spinous cell
Basal cell - Granular cell
Basal cell - Junctional epithelium
Basal cell - Mitotic cell
Basal cell - T cell
Basal cell - B cell
Basal cell - Macrophage
Basal cell - Endothelial cell
Basal cell - Fibroblast
Basal cell - Pericyte
Spinous cell - Basal cell
Spinous cell - Spinous cell
Spinous cell - Granular cell
Spinous cell - Junctional epithelium
Spinous cell - Mitotic cell
Granular cell - Spinous cell
Junctional epithelium - Basal cell
Junctional epithelium - Junctional epithelium
Junctional epithelium - Mitotic cell
Mitotic cell - Basal cell
Mitotic cell - Spinous cell
Mitotic cell - Granular cell
Mitotic cell - Junctional epithelium
Mitotic cell - Mitotic cell
Mitotic cell - Endothelial cell
Y M O
−log10(P)
Mean expression

Figure S5

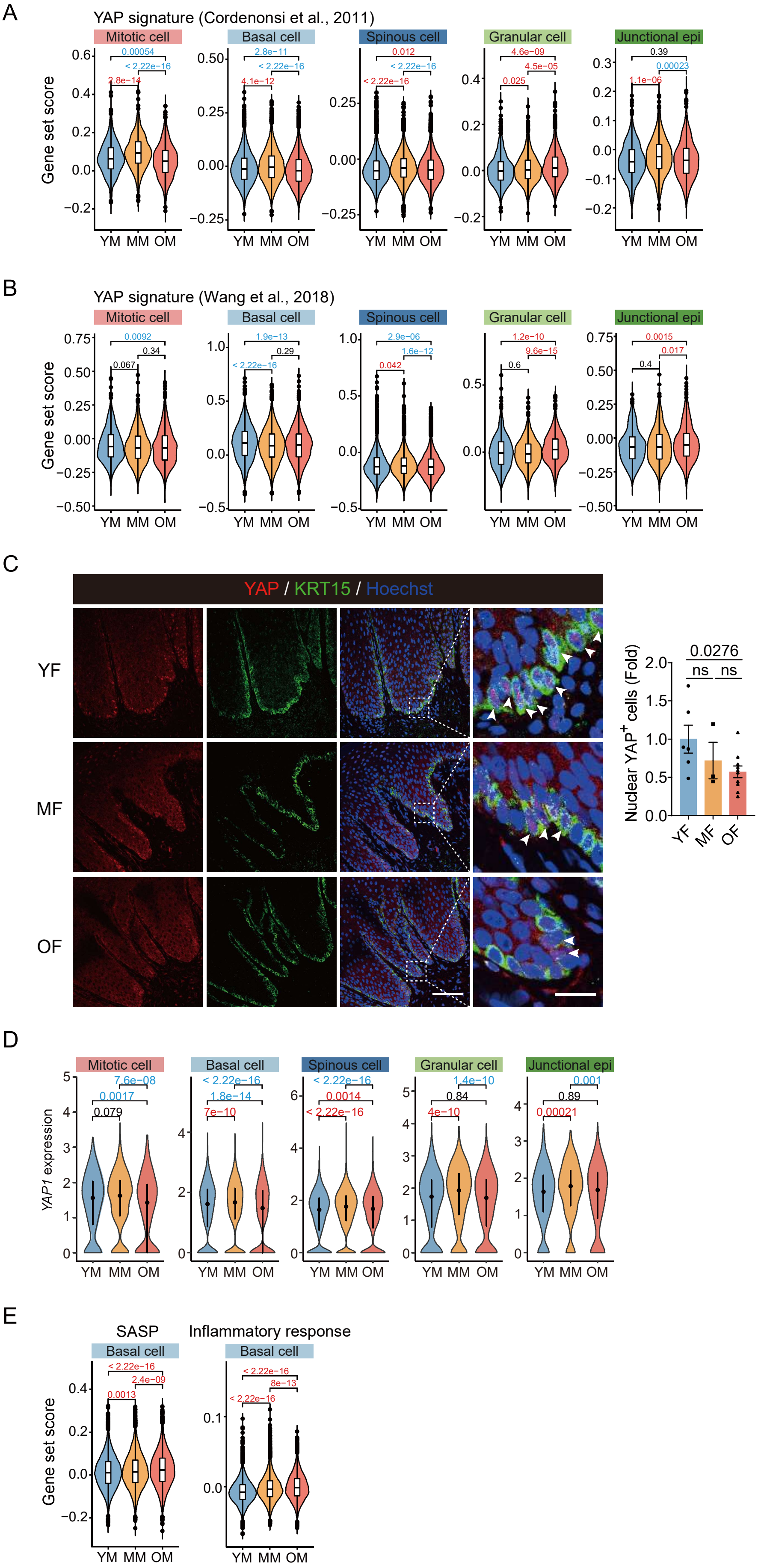
A
YAP signature (Cordenonsi et al., 2011)
Mitotic cell
Basal cell
Spinous cell
Granular cell
Junctional epi
Gene set score
0.00054
< 2.22e−16
2.8e−14
2.8e−11
4.1e−12
0.012
< 2.22e−16
4.6e−09
4.5e−05
0.025
0.39
0.00023
1.1e−06
YM MM OM
B
YAP signature (Wang et al., 2018)
Mitotic cell
Basal cell
Spinous cell
Granular cell
Junctional epi
Gene set score
0.0092
0.34
0.067
1.9e−13
0.29
< 2.22e−16
2.9e−06
1.6e−12
0.042
1.2e−10
9.6e−15
0.6
0.0015
0.017
0.4
YM MM OM
C
YAP / KRT15 / Hoechst
YF
MF
OF
Nuclear YAP+ cells (Fold)
0.0276
ns
ns
YF
MF
OF
D
Mitotic cell
Basal cell
Spinous cell
Granular cell
Junctional epi
YAP1 expression
7.6e−08
0.0017
0.079
< 2.22e−16
1.8e−14
7e−10
< 2.22e−16
0.0014
< 2.22e−16
1.4e−10
0.84
4e−10
0.001
0.89
0.00021
YM MM OM
E
SASP
Inflammatory response
Basal cell
Gene set score
< 2.22e−16
2.4e−09
0.0013
< 2.22e−16
8e−13
< 2.22e−16
YM MM OM

Figure S6

A

No.	Sex	Age (Year)
1	Female	14
2	Female	16
3	Female	21
4	Male	24
5	Male	24
6	Male	26
7	Male	26
8	Female	26
9	Male	27
10	Female	27
11	Female	29
12	Female	37
13	Male	39
14	Female	40
15	Male	46
16	Male	53
17	Male	58
18	Male	70

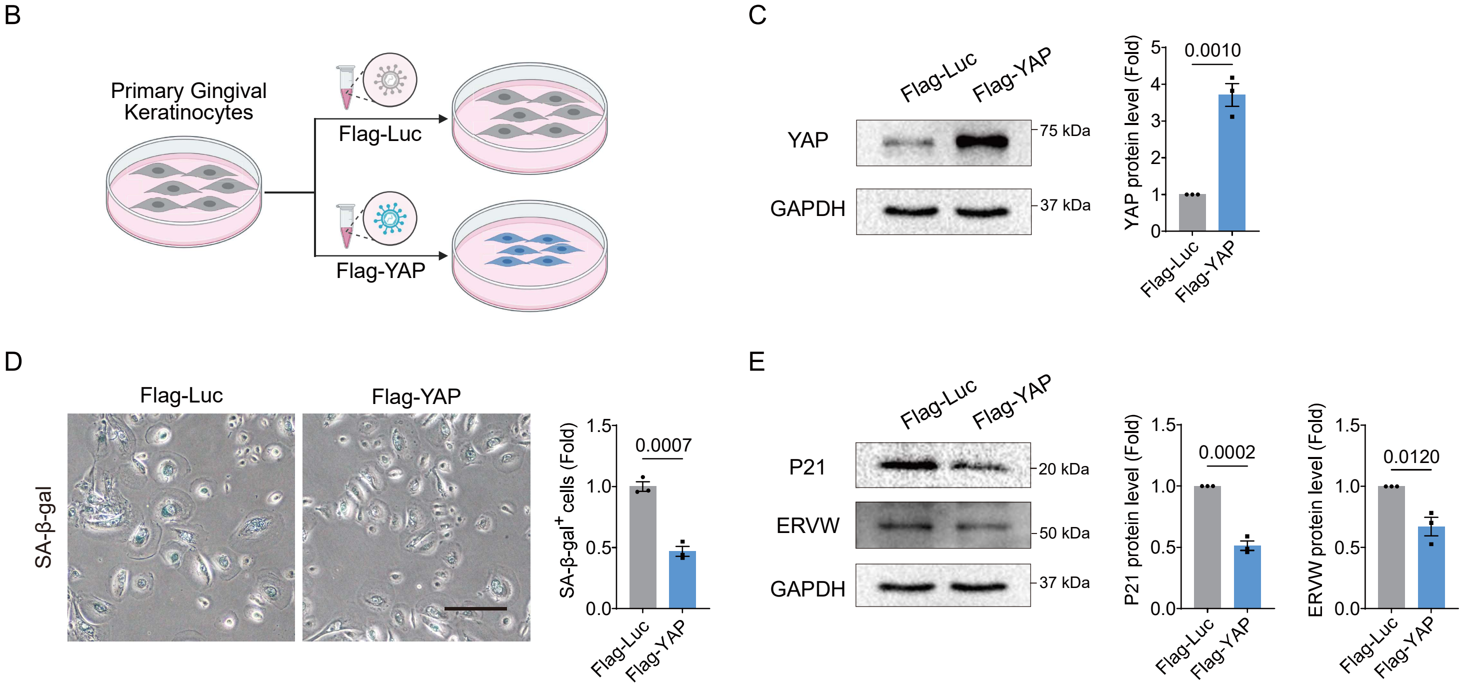

Supplement: pwae017_suppl_Supplementary_Materials [file pwae017_suppl_supplementary_materials.zip › PAC-23559-LGH-Supplementary materials/PAC-23559-LGH-Supplementary materials.pdf]
